# Construction of a novel anaerobic pathway in *Escherichia coli* for propionate production

**DOI:** 10.1186/s12896-017-0354-5

**Published:** 2017-04-14

**Authors:** Jing Li, Xinna Zhu, Jing Chen, Dongdong Zhao, Xueli Zhang, Changhao Bi

**Affiliations:** Key Laboratory of Systems Microbial Biotechnology, Tianjin Institute of Industrial Biotechnology, Chinese Academy of Sciences, Tianjin, 300308 People’s Republic of China

**Keywords:** Propionate, Succinate, Methylmalonyl CoA mutases, Sbm operon, Novel fermentation pathway

## Abstract

**Background:**

Propionate is widely used as an important preservative and important chemical intermediate for synthesis of cellulose fibers, herbicides, perfumes and pharmaceuticals. Biosynthetic propionate has mainly been produced by Propionibacterium, which has various limitations for industrial application.

**Results:**

In this study, we engineered *E. coli* by combining reduced TCA cycle with the native sleeping beauty mutase (Sbm) cycle to construct a redox balanced and energy viable fermentation pathway for anaerobic propionate production. As the cryptic Sbm operon was over-expressed in *E. coli* MG1655, propionate titer reached 0.24 g/L. To increase precursor supply for the Sbm cycle, genetic modification was made to convert mixed fermentation products to succinate, which slightly increased propionate production. For optimal expression of Sbm operon, different types of promoters were examined. A strong constitutive promoter Pbba led to the highest titer of 2.34 g/L. Methylmalonyl CoA mutase from *Methylobacterium extorquens* AM1 was added to strain T110(pbba-Sbm) to enhance this rate limiting step. With optimized expression of this additional Methylmalonyl CoA mutase, the highest production strain was obtained with a titer of 4.95 g/L and a yield of 0.49 mol/mol glucose.

**Conclusions:**

With various metabolic engineering strategies, the propionate titer from fermentation achieved 4.95 g/L. This is the reported highest anaerobic production of propionate by heterologous host. Due to host advantages, such as non-strict anaerobic condition, mature engineering and fermentation techniques, and low cost minimal media, our work has built the basis for industrial propionate production with *E. coli* chassis.

**Electronic supplementary material:**

The online version of this article (doi:10.1186/s12896-017-0354-5) contains supplementary material, which is available to authorized users.

## Background

Propionate is widely used as an important preservative in animal feed and human foods, which is also a chemical intermediate for synthesis of cellulose fibers, herbicides, perfumes and pharmaceuticals [1, 2]. Currently, China has the propionate consumption of 1000 t annually, but production capacity is only about 200 t, far from meeting the actual needs. Propionate is mainly produced from petroleum via oxo-synthesis [3], similar to many other chemical compounds. Due to exhaustion of resources and serious environmental pollution caused by utilization of fossil resources, production of propionate by microbial fermentation from renewable resources has attracted increased attention [4]. Traditionally, Propionibacteria, such as *Propionibacterium freudenreichii* [5], *Propionibacterium acidipropionici* [6–15], and *Propionibacterium thoenii* [16] were used to produce propionate in strict anaerobic condition. Anaerobic fermentation condition was realized by creation of vacuum followed by flushing with pure nitrogen for 3 times, and then the reactor was sealed with a butyl rubber cap in anaerobic chamber. Thus, fermentation by propionibacteria has various limitations, such as nitrogen flux for maintaining anaerobic condition [15], slow growth,costly complex culture media and lack of metabolic engineering tools for strain improvement [17], which make this technology not economically applicable.

**Fig. 1:**
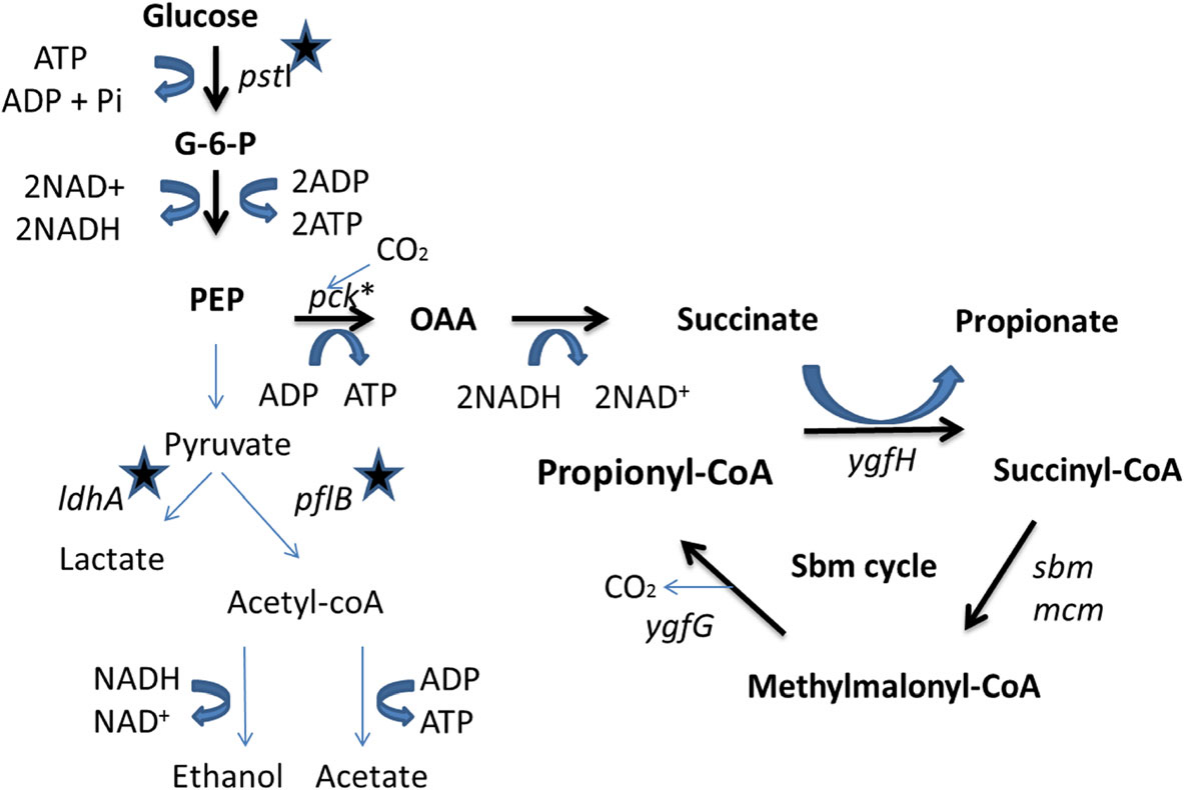
Engineering of an anaerobic propionate fermentation pathway in *E. coli. Bold arrows* indicate engineered pathway; *stars* indicate deleted genes; pck* was a mutated form of the pck in the promoter region to increase its expression

*E. coli* is a potential propionate producer, which carries a cryptic Sbm operon. This operon is constitutively inactivated under natural conditions, which consists of four genes: *sbm*, *ygfD, ygfG* and *ygfH*. These genes encode enzymes that catalyze conversion of succinate to propionate in a cobalamin-dependent metabolic pathway [18]. The Sbm cycle, as illustrated in Fig. 1, includes: Sbm, a methylmalonyl-CoA mutase, which catalyzes rearrangement of succinyl-CoA to methylmalonyl-CoA; YgfG, a methylmalonyl-CoA decarboxylase, which catalyzes decarboxylation of methylmalonyl-CoA to form propionyl-CoA; and YgfH, a propionyl-CoA: succinate-CoA transferase, which transfers CoA group of the propionyl-CoA product to a molecule of succinate, thus primes another round of succinate to propionate decarboxylation. The function of YgfD is unknown, but experiments showed that it might form a functional complex with Sbm [19]. As illustrated in Fig. 1, the function of the cycle is for consumption of succinate. It is most possible that succinate is no longer a major substrate for these *E. coli* strains, so that this operon is silenced during the evolution.

There was only one report we found concerning production of propionate by engineered cell factories [17]. Akawi Lamees et al. knocked out genes involved in glycerol dissimilation (except glpA) to minimize levels of solventogenesis and shift more dissimilated carbon flux toward the C3-fermentative pathway for more flexible redox balancing, and finally achieved propionate titers of more than 11 g/L with yields up to 0.4 g-propionate/g-glycerol in complex fermentation medium and well-controlled micro aerobic condition. And their work was mainly focused on engineering of glycerol utilization [17]. There were also researches concerning products involved Sbm cycle. Zhang et al. studied the role of propionyl-CoA and methylmalonyl-CoA metabolism, and found propionyl-CoA could be supplied for polyketide production by *E. coli* in complex media, but the Sbm cycle might not be directly involved [20]. In the research for propanol production, Sbm cycle was also employed to convert succinyl-CoA to propionyl-CoA. However, due to lack of extensive metabolic network manipulation to convert carbon flux into this pathway and imbalanced ox-reductive pathway design, propanol was low at 150 mg/L in complex medium fermentation [21].

*E. coli* performs mix-acid fermentation when grown anaerobically with acetate, ethanol, lactate, formate and succinate as major products. During fermentation, NADH produced in catabolic process is used to produce fermentation products to regenerate NAD, meanwhile net ATP is produced in the process to sustain cell growth [22]. *E. coli* anaerobic fermentation has many advantages, such as economical fermentation process, minimal cell mass production, and easy genetic manipulation. For these reasons, most commercialized production processes were based on it, for example, industrial production of succinate, L-alanine and D-lactate [23, 24]. By studying reduced TCA cycle and Sbm cycle, we designed a novel anaerobic pathway from glucose to propionate as illustrated in Fig. 1, indicated by bold arrows. This pathway goes through reduced TCA cycle from Phosphoenolpyruvate (PEP) to succinate, which is then converted to propionate by Sbm cycle. In this pathway, NADH production and consumption is balanced, and 3 molecules of ATP can be produced from one glucose, which meets the requirement of a sustainable fermentation pathway. In this study, we attempted to engineer *E. coli* to produce propionate anaerobically with this novel pathway by combining reduced TCA cycle with native Sbm cycle.

## Methods

### Bacterial strains, plasmids and culture media

Strains and plasmids constructed in this study are listed in Table 1. During strain construction, *E. col*i was cultured aerobically at 30 or 37 °C in Luria broth (per liter, 10 g Difco tryptone, 5 g Difco yeast extract, and 5 g NaCl). Ampicillin (100 mg/L), kanamycin (25 mg/L), or chloramphenicol (17 mg/L) was used where appropriate. For multi-plasmid systems, the concentration of each antibiotic was reduced in half to avoid negative impacts on growth.

**Table 1:** Strains and plasmids used in this study

Name	Description, relevant genotype	Source
Strains		
MG1655	Wild type	Lab collection
T110	ATCC 8739ΔldhA ΔpflB ΔptsI Ppck*-galP-pck^a^	[30]
T110ΔducBC	T110 ΔducB ΔducC	[31]
Plasmids		
pET28a		Lab collection
pACYC184		Lab collection
pEC-XK99E		Lab collection
plac-Sbm	Derived from pET28a,P*lai*:: sbm-ygfD-ygfG	This study
ptac-Sbm	Derived from pET28a,P*tac*:: sbm-ygfD-ygfG	This study
pbba-Sbm	Derived from pET28a,P*bba*:: sbm-ygfD-ygfG	This study
plac-ABb	Derived from pACYC184,P*lac*:: mcmA-mcmB-meab	This study
pbba-ABb	Derived from pACYC184,P*bba*:: mcmA-mcmB-meab	This study

^a^Ppck* is a mutated form of the pck promoter (G to A at position 64 relative to the ATG start codon) [30]

### Anaerobic fermentation and production of propionate

For propionate production in batch fermentation, fresh colonies were picked from LB agar plates and inoculated into 15 × 100 mm tubes containing 3 mL LB medium and grown overnight at 37 °C with shaking at 250 rpm. This seed culture was used to inoculate a 10 mL glass screw-cap tube containing 5 mL New Brunswick Scientific (NBS) mineral salts [19] medium with 25 g liter^−1^ glucose, 100 mM potassium bicarbonate, 0.2 μM cyanocobalamin, and 50 mM MOPs (initial OD600 = 0.1). Culture tubes were then incubated at 30 or 37 °C anaerobically with shaking at 250 rpm to increase cell mass. After 4 h, isopropyl-beta- D-thiogalactopyranoside (IPTG) (final concentration: 1 mM) was used to induce gene expression regulated by Plac and Ptac promoters, then cultures were incubated anaerobically. After fermentation, samples were collected to measure propionate titer.

For the experiment of testing the effect of expression of plac-ABb in T110 (pbba-Sbm), different final concentrations of IPTG (0.02, 0.08, 0.2 and 1 mM) were added. For propionate production in mini bioreactors, fresh colonies were picked from NBS mineral salts plates containing 20 g liter^−1^ glucose, inoculated into 100 mL flasks containing 10 mL mineral salts medium with 20 g liter^−1^ glucose, and grown overnight at 37 °C and 250 rpm. Cultures were then transferred to a 250-mL mini bioreactor containing 200 mL mineral salts medium with 20 g liter^−1^ glucose and 100 mM potassium bicarbonate (initial OD600 = 0.1) at 30 or 37 °C. The pH was maintained at 7.0 by automatic addition of 6 M potassium hydrate.

### Genomic DNA and plasmid extraction

The genomic DNA of *E. coli* MG1655 and *M. extorquens* AM1 were extracted using Promega Wizard Genomic DNA Purification Kit (Madison, MI). Plasmids were extracted from *E. coli* using Qiagen QIAprep or MiniPrep plasmid purification kit (Valencia, CA). Experiments were performed according to manufacture protocols.

### Plasmid construction

Plasmids used in this study are listed in Table 1. Three genes *sbm, ygfD,* and *ygfG* from the Sbm operon were PCR-amplified from *E. coli* MG1655 genomic DNA, and cloned to pET28a with their expression under the regulation of inducible promoter Plac or Ptac [25] or constitutive promoter Pbba [26]. To construct plac-Sbm plasmid, Sbm operon was PCR amplified using the c-lac-sbm primer set. Plasmid backbone of pET28a was PCR amplified using the c-lac-pet28a primer set. Plac DNA fragment was PCR amplified using the c-lac-lac primer set. Double terminator DNA fragment was PCR amplified using the c-lac-dbl term primer set. Plasmid backbone of pET28a and DNA fragments were assembled with Golden Gate Assembly [27]. In a 10 μL Golden Gate reaction mixture, 100 ng of the linearized vector backbone and equimolar amounts of the other assembly pieces were blended with 0.5 μL BsaI-HF, 0.5 μL T4 ligase and 1× T4 ligase buffer (New England Biolabs, Ipswich, MA). The reaction was carried out in a thermocycler using the following program: 37 °C for 3 min, 16 °C for 4 min, repeat 1–2 for 25 cycles, 50 °C for 5 min, 80 °C for 5 min, and 4 °C hold.

To construct ptac-Sbm plasmid, tac-Sbm1 was PCR amplified from plac-Sbm using the c-tac-sbm1 primer set with half of Ptac promoter embed in reverse, and the c-tac-sbm2 primer set with the other half of Ptac promoter embed in forward primers. The assembly process was the same as above. Same process was used for the pbba-Sbm plasmid.

The methylmalonyl-CoA mutase genes (mcmA, mcmB, meab) [28] from *M. extorquens* AM1 were cloned into plasmid pACYC184 with their expression under the regulation of Plac or Pbba promoter. To make the construct of plac-ABb, mcmA, mcmB and meab were PCR amplified from *M. extorquens* AM1 genomic DNA using the c- mcmA, c- mcmB and c- meab primer set, whereas the Plac fragment was PCR-amplified from pEC-XK99E using the c- plac-99E primer set. Backbone of pACYC184 and the above four DNA fragments were assembled with Gibson assembly method [29] instead of Golden Gate, due to presence of restriction site of *BsaI*. 100 ng of the linearized vector backbone and equimolar amounts of the other assembly pieces were added to 15 μl Gibson assembly master mix with a 20 μl total volume. The mixture was incubated at 50 °C for 60 min to react. Then 2 μl of the assembly reaction solution was transformed into 50 μl of competent *E. coli* DH5α to obtain successful assembled plasmids. All recombinant plasmids were confirmed by colony PCR and DNA sequencing.

**Fig. 2:**
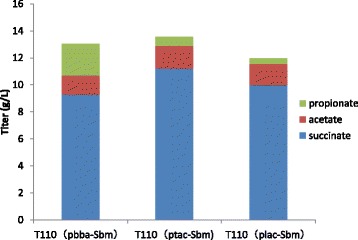
Propionate and other fermentation products of engineered strains with *sbm* operon under different promoters. Strains in glass screw-cap tubes with NBS (20 g/L glucose) media were cultivated aerobically in the dark at 37 °C, until the cultures reached at an OD600 of 0.5. Then cultures were added with appropriate concentrations of IPTG and cultivated anaerobically in dark for 72 h. All experiments were performed in triplicate

To construct pbba-ABb, bba-ABb1 was PCR amplified from plac-ABb using the c-bba-ABb1 primer set with part of Pbba promoter embed in reverse primer, and the c-bba-ABb2 primer set with the rest of Pbba promoter and RBS embed in forward primers. The assembly process was the same as above. Details of the primers above are shown in Additional file 1: Table S4, and plasmid maps with sequences are also included in Additional file 1.

### Bacterial transformation and electroporation

Electroporation of *E. coli* was carried out according to the procedures as follows. Fresh colony was picked from LB agar plates, inoculated into a tube containing 1 mL LB medium and grown overnight at 37 °C with shaking at 250 rpm. This seed culture was used to inoculate a 250-mL flask containing 50 mL LB medium at 37 °C aerobically with shaking at 250 rpm to increase cell mass. Cells at exponential phase were collected and incubated in ice for 30 min. Cells were washed and suspended in chilled 10% glycerol, and 50 μL aliquots of cells were transferred to a 1.5-mL centrifuge tubes. 1 μg of DNA sample was mixed with cells and incubated on ice for 5 min before the mixture was transferred to a chilled 0.1 cm electroporation cuvette (Bio-Rad, Hercules, CA). An electric pulse (18 kV/cm,25 μF capacitance and 200 Ω resistance) was applied to the cuvette for electroporation. Then cells were suspended in 1 mL LB and incubated in a tube at 37 °C for 40 min before plated on LB agar plates containing appropriate antibiotic. The plates were incubated at 37 °C overnight then.

### HPLC analysis

Cell growth was analyzed by measuring the optical density value at 600 nm. For HPLC analyses, culture samples were centrifuged for 3 min at 12,000 × g to recover the supernatant fraction which was filtered with a 0.2 μM syringe filter prior to their injection. Metabolites in fermentation broth were analyzed using HPLC (Agilent Technologies, USA) equipped with an Aminex HPX87 column (BioRad Laboratories, USA) and a refractive index detector (RID, Agilent Technologies, USA). The column temperature was maintained at 65 °C when conducting analysis. The mobile phase was 5 mM H_2_SO_4_ running at 0.5 mL/min [30]. The RID was connected to an integrator (C-R8A, Shimadzu, Kyoto, Japan) for chromatographic data processing. Pure samples of various metabolites with concentrations ranging from 0.02 to 12.0 g/L were used as standards for calibration. Three repeats were performed, and the error bars represented standard deviation.

## Results

### Engineering of an anaerobic propionate fermentation pathway in *E. coli*

A novel anaerobic pathway from glucose to propionate was engineered in *E. coli* by combining reduced TCA cycle with native Sbm cycle, as illustrated in Fig. 1. This pathway goes through reduced TCA cycle from PEP to succinate, which is then converted to propionate by Sbm cycle. Sbm operon from MG1655 was cloned and expressed under control of Plac promoter. A titer of 0.24 g/L propionate was obtained with functionalized Sbm operon, compared to 0.08 g/L by wild type strain, which suggested the fermentation pathway was established. In the novel pathway, immediate precursor of propionate is succinate (Table 2). To increase carbon flux to this route, genetic modifications were made to *E. coli* to eliminate mix-acid fermentation products other than succinate. To be specific, *ldhA* (encodes lactate dehydrogenase) was knocked out to eliminate lactate production; and *pflB* (encodes pyruvate-formate lyase) was knocked out to decrease formate, acetate and ethanol production. *ptsI* was deleted to conserve PEP from carbohydrate phosphotransferase system (PTS); *pck* (encodes PEP carboxykinase) with *galP* promoter was overexpressed to increase substrate utilization efficiency of the reduced TCA cycle. The resulting strain with succinate as major fermentation product was designated as T110 strain [30]. In the engineered strain T110 (plac-Sbm), propionate fermentation production was increased to 0.43 g/L, indicated that precursor supply of propionate pathway was a key factor (Table 2). To increase succinate accumulation inside cell to supply more precursor, two succinate excretion transporters, DucB and DucC [31], were deleted. However, strain T110ΔducBC (plac-Sbm) suffered deficient cell growth, with an OD_600_ of 1.3, compared with T110 (plac-Sbm) with OD_600_ of 4.4. Thus both succinate and propionate production were significantly reduced, which suggested this was not a feasible strategy (Table 2).

**Table 2:** Fermentation profiles of *E. coli* strains engineered with novel propionate fermentation pathway

	Titers (g/L)				Yield (Propionate/Glucose)	
	Succinate	Lactate	Acetate	Propionate	Ethanol	(mol/mol)
MG1655	1.00 ± 0.11	7.12 ± 0.21	2.85 ± 0.06	0.08 ± 0.02	0.67 ± 0.14	0.013 ± 0.002
MG1655 (plac-Sbm)	0.28 ± 0.07	1.91 ± 0.34	3.12 ± 0.74	0.24 ± 0.02	0.61 ± 0.25	0.039 ± 0.005
T110	10.99 ± 0.18	0	2.62 ± 0.19	0.01 ± 0.01	0	0
T110 (plac-Sbm)	9.98 ± 0.12	0	1.56 ± 0.09	0.43 ± 0.07	0	0.068 ± 0.002
T110ΔducBC	2.85 ± 0.25	0	1.48 ± 0.15	0.008 ± 0.005	0	0
T110ΔducBC (plac-Sbm)	1.45 ± 0.31	0	1.09 ± 0.27	0.13 ± 0.06	0	0.021 ± 0.005

Strains in glass screw-cap tubes were cultivated aerobically in the dark at 37 °C, until the cultures reached an OD600 of 0.5. Then culture was added with 1 mM IPTG and cultivated anaerobically in the dark for 72 h. All experiments were performed in triplicate

Methylmalonyl-CoA mutase activity requires presence of cyanocobalamin [32–34]. Since cyanocobalamin degrades when exposed to light, the fermentation process was carried out in dark. To determine the optimal supplementation of cyanocobalamin, various concentrations were added for propionate fermentation. With the propionate production strain T110 (plac-Sbm), 0.2 μM was determined to be the optimal cyanocobalamin supplementation concentration (Additional file 1: Figure S1 and Table S1).

### Modulation of Sbm operon expression for enhanced propionate production

In T110 (plac-Sbm) fermentation, propionate titer was 0.43 g/L, much lower than the 9.98 g/L succinate, which suggested inability of current Sbm cycle to convert precursor to final product. To increase expression of Sbm operon, thus increase efficiency of Sbm cycle, Plac promoter was replaced with either Ptac or a strong constitutive Pbba promoter, resulting in strain T110 (ptac-Sbm) and T110 (pbba-Sbm). While Propionate production of T110 (ptac-Sbm) only slightly increased compared with starting strain T110 (plac-Sbm), T110 (pbba -Sbm) had significant improvement to reach 2.34 g/L (Fig. 2 and Additional file 1: Table S2). This result indicated employment of a strong constitutive promoter successfully increased Sbm operon expression and improved Sbm cycle efficiency to convert more succinate to propionate. In this way, more carbon flux was diverted to the novel propionate fermentation pathway.

### Supplementation and modulation of heterologous Methylmalonyl CoA mutase for increased propionate production

**Fig. 3:**
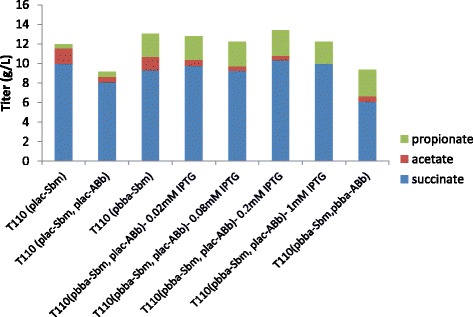
The effects of expression of *M. extorquens* AM1 Methylmalonyl CoA mutase on propionate production. Strains were cultivated aerobically in glass screw-cap tubes in dark at 37 °C, until the cultures reached at an OD600 of 0.5. Then IPTG with appropriate concentration was added and cells were cultivated anaerobically in the dark for 72 h at 30 °C. All experiments were performed in triplicate

The reversible step of succinyl-CoA converting to methylmalonyl-CoA was reported to be a possible rate limiting step in propionate fermentation pathway, probably due to instability of *E. coli* Sbm [18]. This enzyme was found in both bacterial and animal cells, such as *P. shermanii*, *M. extorquens* AM1 [35], human, and mice. Since methylmalonyl CoA mutase of *P. shermanii* was reported to be inactivate in aerobic conditions [28], we chose this enzyme from aerobic bacteria *M. extorquens* AM1 in this research. Methylmalonyl CoA mutase from *M. extorquens* AM1 has two subunits, mcmA and mcmB. Meab is an associate protein, forms a complex with methylmalonyl-CoA mutase and stimulates in vitro mutase activity [28]. The *ABb* operon encodes these three proteins. To increase catalytic activity of this step, operon *ABb* was expressed with control of Plac promoter and supplemented to T110 (plac-Sbm). Different concentrations of IPTG were used to modulate expression of *ABb* to found optimal condition. It was found that propionate production increased with higher concentrations of IPTG, except at the highest level of 1 mM (Fig. 3, Additional file 1: Table S3). The general trend of increased production with higher induction concentrations suggested higher expression level of *ABb* improved propionate production. So that, the constitutive strong promoter Pbba was used to express this enzyme complex. As a result, the resulting strain T110 (pbba-sbm, pbba-ABb) achieved the highest production titer of 2.72 g/L in screw-cap tube fermentation.

To better evaluate the strains with propionate fermentation pathway, 250 mL mini bioreactors were used for fermentation. As illustrated in Table 3, propionate production generally increased compared with in 10 mL screw-cap tubes. Propionate production of our best strain, T110 (pbba-sbm, pbba-ABb) reached 4.95 g/L and yield reached 0.49 mol/mol from glucose. Based on molar amount, the final titer of succinate and propionate was similar at around 0.07 M, indicated that half of succinate was converted by Sbm cycle to propionate in the fermentation process. At this point, the novel propionate fermentation pathway was successfully established as a major pathway in engineered strain, even though future efforts are still necessary to make propionate the sole fermentation product.

**Table 3:** Propionate production of engineered strains in NBS (20 g/L glucose) media with 250 mL mini bioreactors

	Titer (g/L)				Yield (Propionate/Glucose)	
	Succinate	Acetate	Propionate	Ethanol	(g/g)	(mol/mol)
T110 (plac-Sbm)	15.49 ± 0.05	2.36 ± 0.12	0.49 ± 0.21	0.75 ± 0.08	0.02 ± 0.009	0.05 ± 0.02
T110 (pbba-Sbm)	9.27 ± 0.02	0.49 ± 0.14	4.75 ± 0.5	0	0.19 ± 0.02	0.48 ± 0.05
T110 (pbba-Sbm, plac-ABb)	6.10 ± 0.21	0.38 ± 0.08	3.05 ± 0.12	0	0.19 ± 0.005	0.47 ± 0.01
T110 (pbba-Sbm, pbba-ABb)	8.48 ± 0.20	0.65 ± 0.11	4.95 ± 0.08	0	0.20 ± 0.003	0.49 ± 0.007

Strains were cultivated aerobically in the dark in 250 mL mini bioreactors in NBS (20 g/L glucose) media at 37 °C, until the cultures reached at an OD600 of 0.5. Then IPTG with appropriate concentration was added and cultivated anaerobically in the dark for 72 h at 30 °C, for methylmalonyl-CoA mutase from *M. extorquens* AM1 was active at 30 °C. All experiments were performed in triplicate

## Discussions

Three metabolic engineering strategies were successfully designed and applied in this work, including: activation of *E. coli* native cryptic pathway, multi enzymes to enhance efficiency of one reaction in a pathway, and construction of a novel anaerobic pathway with balanced ox-reduction status and adequate energy production.

All biological systems are capable of short term response to environmental changes and, on longer time scales, to evolutionary adaptation. It was suggested that cryptic or latent pathways are consequences of such adaption. By inactivate once active pathways, microbes are able to save materials and energy associated with expression of the genes, and to change metabolic network for adaption of new environment [36, 37]. Cryptic or latent pathway activation is a classic subject in metabolic engineering, mainly for production of therapeutic natural products in microorganisms such as *Aspergillus, Streptomyces,* or *Pseudomona*s [38]*.* However, only a few examples can be found with application in metabolic engineering for the most popular chassis *E. coli,* very few of which was directly for novel product synthesis [39, 40]. Theoretically, native genes guarantee a better expression status compared to heterologous ones, since they were once expressed and functioning in the system. This was the reason we planned to employ the original Sbm cycle instead of heterologous pathways from natural propionate producers, which was proved to be a successful strategy. Thus, utilizing native cryptic pathways should be given more attention in metabolic engineering researches, since such strategies might be a better choice than normal heterologous gene expression strategies.

During past years, our team was able to engineer a few efficient microbial cell factories to produce L-alanine, L-lactic acid or succinate, for which the general strategy was establishment of an balanced and sustainable metabolic pathway for anaerobic fermentation [23, 24]. In such a balanced fermentation pathway, NADH produced in catabolic process is used to produce fermentation products to regenerate NAD, meanwhile net ATP should be produced in the process to sustain cell growth [22]. When microbes were engineered to rely on a balanced fermentation pathway, the host has to produce the end product of the pathway to live. Thus, target product is coupled with cell growth [21]. Then many rational or irrational metabolic engineering strategies can be applied to such microbes for construction of a microbial cell factories. Anaerobic fermentation also has many advantages for industrial scale fermentation, such as no necessary for oxygen supplementation and minimal cell mass production, which minimizes process and equipment cost. For these reasons, the three fermentation producers we have constructed have all been commercialized to produce biochemical products. The propionate pathway we constructed in this work is ox-reduction balanced. In this pathway, NADH production and consumption is balanced, and 3 molecules of ATP is produced from one glucose, which meets the requirement of a sustainable fermentation pathway as illustrated in Fig. 1. Significant production of propionate was achieved with this strategy, which proved balancing fermentation pathway a key metabolic engineering strategies worth serious consideration.

We have a few plans to be carried out in near future to further improved propionate production. The major problem left is co-production of succinate in current best strain, which is a major intermediate and the substrate for Sbm cycle. Strategies should be developed to push the pathway towards the end product propionate. One strategy we attempted in this work was to eliminate excretion of succinate by deletion of succinate transporter genes *ducB* and *ducC*, hoping for increased internal concentration of succinate, which is the precursor of propionate (Fig. 1). Thus the reaction balance was pushed towards propionate production. However, deletion of succinate transporter genes caused significant growth defect probably due to increased internal accumulation of succinate, which was not converted into propionate in time and might be toxic. Balancing succinate precursor supply and Sbm pathway activity is a major problem to be solved. Accumulation of internal propionate for lack of an efficient propionate transporter might also be another problem. Thus, search of efficient propionate exporters might be a feasible strategy. For hyper lactic acid production, industrial solution was to accumulate it with Ca^2+^, forming insoluble compound to pull the reaction balance toward the end product. Such biochemical strategy might be our solution to specifically accumulate insoluble propionate salts for higher production.

There was only one report we found concerning production of propionate by engineered cell factories [17]. Akawi Lamees et al. achieved production titers of 11 g/L with yields up to 0.4 g-propionate/g-glycerol. Different from our strategy to evolve a balanced fermentation pathway with elaborated metabolic engineering strategies, their work was mainly focused on engineering of substrate utilization of glycerol. And the production titer was obtained using complex fermentation medium supplemented with 10 g/L yeast extract in well-controlled micro aerobic condition bioreactor. The fermentation result in our work was achieved in anaerobic condition using an inexpensive NBS mineral salt medium.

## Conclusions

**Fig. 4:**
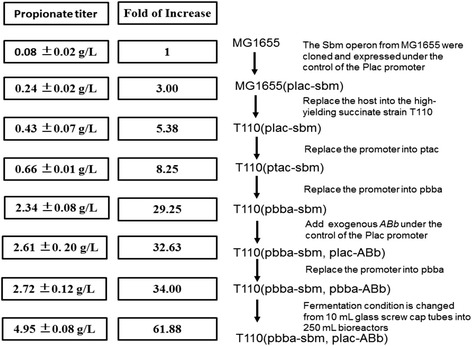
Improving production of propionate by a series of engineering strategies and manipulations

In this study, we established a novel fermentation pathway *E. coli* by combining reduced TCA cycle with the native Sbm cycle for production of propionate anaerobically. As illustrated in Fig. 4, with various metabolic engineering strategies, the propionate titer from fermentation was increased step-by-step of 61.88 fold and reached 4.95 g/L. This is the reported highest anaerobic production of propionate by heterologous host. Due to host advantages, such as non-strict anaerobic condition, mature engineering and fermentation technique, and low cost minimal media, our work has built the basis for industrial propionate production with *E. coli* chassis.

## Additional file

Additional file 1: Figure S1. Propionate production of strain T110 (plac-Sbm) with various concentration of cyanocobalamin. **Table S1.** Propionate production of strain T110 (plac-Sbm) with various concentration of cyanocobalamin. **Table S2.** Propionate and fermentation products of engineered strains with sbm operon under different promoters. **Table S3.** The effects of expression of *M. extorquens* AM1 Methylmalonyl CoA mutase on propionate production. **Table S4.** PCR primers used in this work. **Plasmid and sequence data.** (PDF 756 kb)

## Abbreviations

*ABb*: mcmA, mcmB and meab
Amp: Ampicillin
Cm: Chloramphenicol
IPTG: Isopropyl β-d-thiogalactoside
Kan: Kanamycin
*M. extorquens*AM1: *Methylobacterium extorquens* AM1
OAA: Oxaloacetate
PEP: Phosphoenolpyruvate
PTS: Phosphotransferase system
Sbm: Sleeping beauty mutase
