# Supplementary material for: Construction of a novel anaerobic pathway in Escherichia coli for propionate production
Source: BMC Biotechnol. 2017 Apr 14;17:38. doi: 10.1186/s12896-017-0354-5 (PMC5391575; doi:10.1186/s12896-017-0354-5)
Supplement: Additional file 1: Figure S1. — Propionate production of strain T110 (plac-Sbm) with various concentration of cyanocobalamin. Table S1. Propionate production of strain T110 (plac-Sbm) with various concentration of cyanocobalamin. Table S2. Propionate and fermentation products of engineered strains with sbm operon under different promoters. Table S3. The effects of expression of M. extorquens AM1 Methylmalonyl CoA mutase on propionate production. Table S4. PCR primers used in this work. Plasmid and sequence data. (PDF 756 kb) [file 12896_2017_354_MOESM1_ESM.pdf]

Applied Microbiology and Biotechnology

Construction of a novel anaerobic pathway in *Escherichia coli* for propionate production

Jing Li[1], Xinna Zhu[1], Jing Chen[1], Dongdong Zhao[1], Xueli Zhang[1]* and Changhao Bi[1]*

Tianjin Institute of Industrial Biotechnology, Chinese Academy of Sciences, Tianjin 300308, P R China

1 Key Laboratory of Systems Microbial Biotechnology, Tianjin Institute of Industrial Biotechnology, Chinese Academy of Sciences

* Corresponding authors

Mailing address:

Tianjin Institute of Industrial Biotechnology, Chinese Academy of Sciences, Tianjin 300308, P R China

Key Laboratory of Systems Microbial Biotechnology, Chinese Academy of Sciences, Tianjin 300308, P R China.

E-mail: zhang_xl@tib.cas.cn; bi_ch@tib.cas.cn

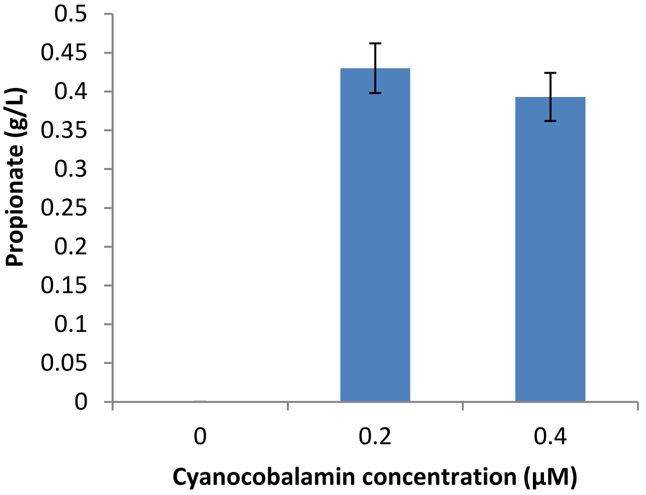

Figure S1. propionate production of strain T110 (plac-Sbm) with various concentration of cyanocobalamin. Propionate production was dependent on supplementation of cyanocobalamin, and titer of propionate reached peak value at concentrations of 0.2 μM. Strains were cultivated micro-aerobically in the dark at 37°C, until the cultures reached at an OD600 of 0.5. Then culture was added IPTG (1 mM) with appropriate concentration and cultivated anaerobically in dark for 72 h. All experiments were performed in triplicate.

Table S1. propionate production of strain T110 (plac-Sbm) with various concentration of cyanocobalamin

g/L	propionate	propionate	propionate
cyanocobalamin (μM)	0	0.2	0.4
average value	0	0.430	0.393
standard deviation	0	0.032	0.031

Table S2. Propionate and fermentation products of engineered strains with sbm operon under different promoters. Strains in glass screw-cap tubes with NBS (20 g/L glucose) media were cultivated micro-aerobically in the dark at 37°C, until the cultures reached at an O.D600 of 0.5. Then added IPTG with appropriate concentration and cultivated anaerobically in the dark for 72 h. All experiments were performed in triplicate.

titer (g/L)	Succinate	Acetate	propionate
T110（pbba-Sbm）	9.30±0.18	1.39±0.12	2.34±0.08
T110（ptac-Sbm)	11.21±0.12	1.68±0.11	0.66±0.01
T110（plac-Sbm）	9.99±0.12	1.56±0.09	0.43±0.07

Table S3. The effects of expression of *M. extorquens* AM1 Methylmalonyl CoA mutase on propionate production. Strains were cultivated micro-aerobically in glass screw-cap tubes in dark at 37°C, until the cultures reached at an OD600 of 0.5. Then IPTG with appropriate concentration was added and cultivated anaerobically in the dark for 72 h at 30°C. All experiments were

performed in triplicate.

titer (g/L)	succinate	acetate	propionate
T110 (pbba-Sbm)	9.30±0.18	1.39±0.12	2.34±0.08
T110(pbba-Sbm, plac-ABb)- 0.02mM IPTG	9.76±0.19	0.64±0.17	2.39±0.21
T110(pbba-Sbm, plac-ABb)- 0.08mM IPTG	9.26±0.13	0.47±0.07	2.51±0.18
T110(pbba-Sbm, plac-ABb)- 0.2mM IPTG	10.32±0.11	0.48±0.08	2.61±0.20
T110(pbba-Sbm, plac-ABb)- 1mM IPTG	9.98±0.13	0	2.26±0.19
T110(pbba-Sbm,pbba-ABb)	6.09±0.08	0.53±0.06	2.72±0.12

Table S4. PCR primers used in this work

**Primers**	Sequence (5′ → 3′)	Sources
c-lac-pet28a	ACCAGGTCTCACGGTCCGCTCATGAATTAATTCTTAGAAAAACTCATCGA; ACCAGGTCTCAGAGCACCGGGATCTCGACCG	This study
c-lac-lac	ACCAGGTCTCAGCTCCTTGGCGGCAAACTTACATTAATTGC; ACCAGGTCTCATTAGGATGGCCTCCTTTGAATTCAATTGTTATCCGC	This study
c-lac-sbm	ACCAGGTCTCACTAATGTCTAACGTGCAGGAGTGGC; ACCAGGTCTCAATGACGAGTCATTAACCCAGCATCGAGC	This study
c-lac-dbl term	ACCAGGTCTCATCATCTCCAGGCATCAAATAAAACGAAAGGC; ACCAGGTCTCAACCGCAGCCGAACGCCCTAG	This study
c-tac-sbm1	CACACCAGGTCTCAAATCCCTGCGAACGAAGGAGTAAAAATGG; CACACCAGGTCTCACGAGCCGATGATTAATTGTCAAAACGCAATTAATGTAAGTTTGCCGCC	This study
c-tac-sbm2	CACACCAGGTCTCACTCGTATAATGTGTGGAATTGTGATTGAATTCAAAGGAGGCCATCC; CACACCAGGTCTCAGATTAATGACCAACGAAATTAGGTTTACG	This study
c-bba-sbm1	CACACCAGGTCTCAAATCCCTGCGAACGAAGGAGTAAAAATGG; CACACCAGGTCTCATAGGACTGAGCTAGCTAACGCAATTAATGTAAGTTTGCCGCC	This study
c-bba-sbm2	CACACCAGGTCTCACCTAGGTATTATGCTAGCATTGAATTCAAAGGAGGCCATCC; CACACCAGGTCTCAGATTAATGACCAACGAAATTAGGTTTACG	This study
c-pACYC184	TGAGCCGTCTGCTCTCCAGGCATCAAATAAAACGAAAG	This study

	GC; GCTTGTCTGTAAGCGGTGAGAATTACAACTTATATCGTATGGGGC	
c-lac-99E	AGTTGTAATTCTCACCGCTTACAGACAAGCTGTGACCGT; GCTCATCGTGCGTCTGACGAAATTCCTTTTTAACGTTCACTGTTTCCTGTGT	This study
c-mcmA	CGTTAAAAAGGAATTTCGTCAGACGCACGATGAGCTCCCG; GCGACAACCGACTTTACTGGAAAAGCGGACTACTCGGCC	This study
c-mcmB	GTCCGCTTTTCCAGTAAAGTCGGTTGTCGCCTGCCC; ATGGCCCCTTCATTCTGGATGCCGTAGCGATAGGCC	This study
c-meab	TACGGCATCCAGAATGAAGGGGCCATGAGCGCGA; TTGATGCCTGGAGAGCAGACGGCTCACAACCCG	This study
c-bba-ABb1	GATCGGGTTGTGACTCCAGGCATCAAATAAAACGAAAGGC; GGCCTCCTTTGAATTCAATGCTAGCATAATACCTAGGACTGAGCTAGCTGTGAGAATTACAACTTATATCGTATGGGGC	This study
c-bba-ABb2	CAGTCCTAGGTATTATGCTAGCATTGAATTCAAAGGAGGCCATCCTAATGAGCTCCCGCATCCCCG; GTTTTATTTGATGCCTGGAGTCACAACCCGATCAGGGTCGCG	This study

Plasmid and sequence data

plac-Sbm

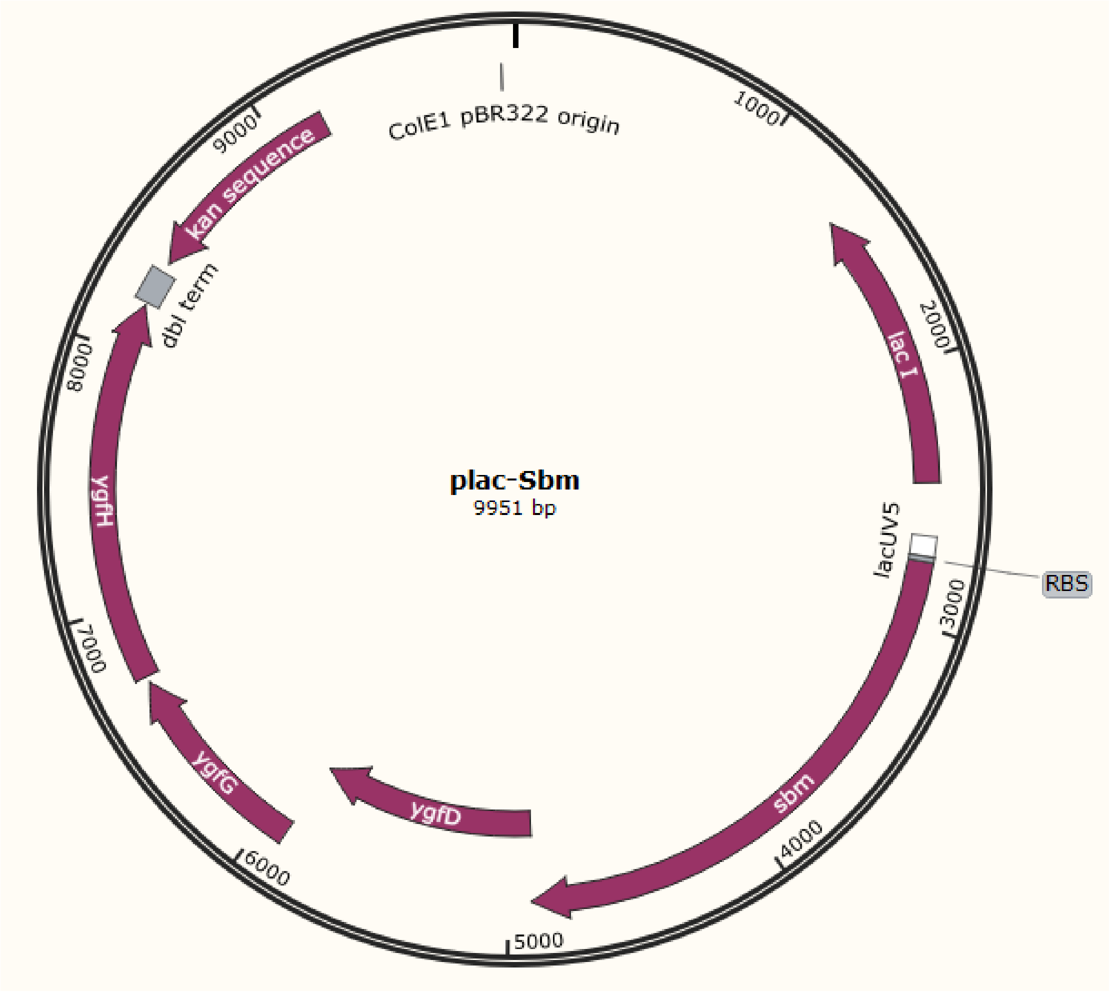

plac-Sbm

>

tttgctcacatgttctttcctgcgttatcccctgattctgtggataaccgtattaccgcctttgagtgagctgataccgctcgccgcagccgaacga
ccgagcgcagcgagtcagtgagcgaggaagcggaagagcgcctgatgcggtattttctccttacgcatctgtgcggtatttcacaccgcatat
atggtgcactctcagtacaatctgctctgatgccgcatagttaagccagtatacactccgctatcgctacgtgactgggtcatggctgcgccccg
acacccgccaacacccgctgacgcgccctgacgggcttgtctgctcccggcatccgcttacagacaagctgtgaccgtctccgggagctgc
atgtgtcagaggttttcaccgtcatcaccgaaacgcgcgaggcagctgcggtaaagctcatcagcgtggtcgtgaagcgattcacagatgtct
gcctgttcatccgcgtccagctcgttgagtttctccagaagcgttaatgtctggcttctgataaagcgggccatgttaagggcggtttttttcctgttt
ggtcactgatgcctccgtgtaagggggatttctgttcatgggggtaatgataccgatgaaacgagagaggatgctcacgatacgggttactgat
gatgaacatgcccggttactggaacgttgtgagggtaaacaactggcggtatggatgcggcgggaccagagaaaaatcactcagggtcaat
gccagcgcttcgttaatacagatgtaggtgttccacagggtagccagcagcatcctgcgatgcagatccggaacataatggtgcagggcgct
gacttccgcgtttccagactttacgaaacacggaaaccgaagaccattcatgttgttgctcaggtcgcagacgttttgcagcagcagtcgcttca
cgttcgctcgcgtatcggtgattcattctgctaaccagtaaggcaaccccgccagcctagccgggtcctcaacgacaggagcacgatcatgc
gcacccgtggggccgccatgccggcgataatggcctgcttctcgccgaaacgtttggtggcgggaccagtgacgaaggcttgagcgaggg
cgtgcaagattccgaataccgcaagcgacaggccgatcatcgtcgcgctccagcgaaagcggtcctcgccgaaaatgacccagagcgctg
ccggcacctgtcctacgagttgcatgataaagaagacagtcataagtgcggcgacgatagtcatgccccgcgcccaccggaaggagctgac
tgggttgaaggctctcaagggcatcggtcgagatcccggtgctccttggcggcaaacttacattaattgcgttgcgctcactgcccgctttccag

tcgggaaacctgtcgtgccagctgcattaatgaatcggccaacgcgcggggagaggcggtttgcgtattgggcgccagggtggtttttcttttc
accagtgagacgggcaacagctgattgcccttcaccgcctggccctgagagagttgcagcaagcggtccacgctggtttgccccagcaggc
gaaaatcctgtttgatggtggttaacggcgggatataacatgagctgtcttcggtatcgtcgtatcccactaccgagatatccgcaccaacgcgc
agcccggactcggtaatggcgcgcattgcgcccagcgccatctgatcgttggcaaccagcatcgcagtgggaacgatgccctcattcagcat
ttgcatggtttgttgaaaaccggacatggcactccagtcgccttcccgttccgctatcggctgaatttgattgcgagtgagatatttatgccagcc
agccagacgcagacgcgccgagacagaacttaatgggcccgctaacagcgcgatttgctggtgacccaatgcgaccagatgctccacgcc
cagtcgcgtaccgtcttcatgggagaaaataatactgttgatgggtgtctggtcagagacatcaagaaataacgccggaacattagtgcaggc
agcttccacagcaatggcatcctggtcatccagcggatagttaatgatcagcccactgacgcgttgcgcgagaagattgtgcaccgccgcttt
acaggcttcgacgccgcttcgttctaccatcgacaccaccacgctggcacccagttgatcggcgcgagatttaatcgccgcgacaatttgcga
cggcgcgtgcagggccagactggaggtggcaacgccaatcagcaacgactgtttgcccgccagttgttgtgccacgcggttgggaatgtaa
ttcagctccgccatcgccgcttccactttttcccgcgttttcgcagaaacgtggctggcctggttcaccacgcgggaaacggtctgataagaga
caccggcatactctgcgacatcgtataacgttactggtttcacattcaccaccctgaattgactctcttccgggcgctatcatgccataccgcgaa
aggttttgcgccattcgatggtgtccgggatctcgacgctctcccttatgcgactcctgcattaggaagcagcccagtagtaggttgaggccgtt
gagcaccgccgccgcaaggaatggtgcatgcaaggagatggcgcccaacagagcttccgatggcgcgccgagaggctttacactttatgct
tccggctcgtataatgtgtggaattgtgagcggataacaattgaattcaaaggaggccatcctaatgtctaacgtgcaggagtggcaacagctt
gccaacaaggaattgagccgtcgggagaaaactgtcgactcgctggttcatcaaaccgcggaagggatcgccatcaagccgctgtataccg
aagccgatctcgataatctggaggtgacaggtacccttcctggtttgccgccctacgttcgtggcccgcgtgccactatgtataccgcccaacc
gtggaccatccgtcagtatgctggtttttcaacagcaaaagagtccaacgctttttatcgccgtaacctggccgccgggcaaaaaggtctttccg
ttgcgtttgaccttgccacccaccgtggctacgactccgataacccgcgcgtggcgggcgacgtcggcaaagcgggcgtcgctatcgacac
cgtggaagatatgaaagtcctgttcgaccagatcccgctggataaaatgtcggtttcgatgaccatgaatggcgcagtgctaccagtactggc
gttttatatcgtcgccgcagaagagcaaggtgttacacctgataaactgaccggcaccattcaaaacgatattctcaaagagtacctctgccgc
aacacctatatttacccaccaaaaccgtcaatgcgcattatcgccgacatcatcgcctggtgttccggcaacatgccgcgatttaataccatcag
tatcagcggttaccacatgggtgaagcgggtgccaactgcgtgcagcaggtagcatttacgctcgctgatgggattgagtacatcaaagcag
caatctctgccggactgaaaattgatgacttcgctcctcgcctgtcgttcttcttcggcatcggcatggatctgtttatgaacgtcgccatgttgcgt
gcggcacgttatttatggagcgaagcggtcagtggatttggcgcacaggacccgaaatcactggcgctgcgtacccactgccagacctcag
gctggagcctgactgaacaggatccgtataacaacgttatccgcaccaccattgaagcgctggctgcgacgctgggcggtactcagtcactg
cataccaacgcctttgacgaagcgcttggtttgcctaccgatttctcagcacgcattgcccgcaacacccagatcatcatccaggaagaatcag
aactctgccgcaccgtcgatccactggccggatcctattacattgagtcgctgaccgatcaaatcgtcaaacaagccagagctattatccaaca
gatcgacgaagccggtggcatggcgaaagcgatcgaagcaggtctgccaaaacgaatgatcgaagaggcctcagcgcgcgaacagtcgc
tgatcgaccagggcaagcgtgtcatcgttggtgtcaacaagtacaaactggatcacgaagacgaaaccgatgtacttgagatcgacaacgtg
atggtgcgtaacgagcaaattgcttcgctggaacgcattcgcgccacccgtgatgatgccgccgtaaccgccgcgttgaacgccctgactca
cgccgcacagcataacgaaaacctgctggctgccgctgttaatgccgctcgcgttcgcgccaccctgggtgaaatttccgatgcgctggaag
tcgctttcgaccgttatctggtgccaagccagtgtgttaccggcgtgattgcgcaaagctatcatcagtctgagaaatcggcctccgagttcgat
gccattgttgcgcaaacggagcagttccttgccgacaatggtcgtcgcccgcgcattctgatcgctaagatgggccaggatggacacgatcg
cggcgcgaaagtgatcgccagcgcctattccgatctcggtttcgacgtagatttaagcccgatgttctctacacctgaagagatcgcccgcctg
gccgtagaaaacgacgttcacgtagtgggcgcatcctcactggctgccggtcataaaacgctgatcccggaactggtcgaagcgctgaaaa
aatggggacgcgaagatatctgcgtggtcgcgggtggcgtcattccgccgcaggattacgccttcctgcaagagcgcggcgtggcggcgat
ttatggtccaggtacacctatgctcgacagtgtgcgcgacgtactgaatctgataagccagcatcatgattaatgaagccacgctggcagaaa
gtattcgccgcttacgtcagggtgagcgtgccacactcgcccaggccatgacgctggtggaaagccgtcacccgcgtcatcaggcactaagt
acgcagctgcttgatgccattatgccgtactgcggtaacaccctgcgactgggcgttaccggcacccccggcgcggggaaaagtacctttctt
gaggcctttggcatgttgttgattcgagagggattaaaggtcgcggttattgcggtcgatcccagcagcccggtcactggcggtagcattctcg
gggataaaacccgcatgaatgacctggcgcgtgccgaagcggcgtttattcgcccggtaccatcctccggtcatctgggcggtgccagtcag
cgagcgcgggaattaatgctgttatgcgaagcagcgggttatgacgtagtgattgtcgaaacggttggcgtcgggcagtcggaaacagaagt
cgcccgcatggtggactgttttatctcgttgcaaattgccggtggcggcgatgatctgcagggcattaaaaaagggctgatggaagtggctgat

ctgatcgttatcaacaaagacgatggcgataaccataccaatgtcgccattgcccggcatatgtacgagagtgccctgcatattctgcgacgta
aatacgacgaatggcagccacgggttctgacttgtagcgcactggaaaaacgtggaatcgatgagatctggcacgccatcatcgacttcaaa
accgcgctaactgccagtggtcgtttacaacaagtgcggcaacaacaatcggtggaatggctgcgtaagcagaccgaagaagaagtactga
atcacctgttcgcgaatgaagatttcgatcgctattaccgccagacgcttttagcggtcaaaaacaatacgctctcaccgcgcaccggcctgcg
gcagctcagtgaatttatccagacgcaatattttgattaaaggaatttttatgtcttatcagtatgttaacgttgtcactatcaacaaagtggcggtca
ttgagtttaactatggccgaaaacttaatgccttaagtaaagtctttattgatgatcttatgcaggcgttaagcgatctcaaccggccggaaattcg
ctgtatcattttgcgcgcaccgagtggatccaaagtcttctccgcaggtcacgatattcacgaactgccgtctggcggtcgcgatccgctctcct
atgatgatccattgcgtcaaatcacccgcatgatccaaaaattcccgaaaccgatcatttcgatggtggaaggtagtgtttggggtggcgcattt
gaaatgatcatgagttccgatctgatcatcgccgccagtacctcaaccttctcaatgacgcctgtaaacctcggcgtcccgtataacctggtcgg
cattcacaacctgacccgcgacgcgggcttccacattgtcaaagagctgatttttaccgcttcgccaatcaccgcccagcgcgcgctggctgt
cggcatcctcaaccatgttgtggaagtggaagaactggaagatttcaccttacaaatggcgcaccacatctctgagaaagcgccgttagccatt
gccgttatcaaagaagagctgcgtgtactgggcgaagcacacaccatgaactccgatgaatttgaacgtattcaggggatgcgccgcgcggt
gtatgacagcgaagattaccaggaagggatgaacgctttcctcgaaaaacgtaaacctaatttcgttggtcattaatccctgcgaacgaaggag
taaaaatggaaactcagtggacaaggatgaccgccaatgaagcggcagaaattatccagcataacgacatggtggcatttagcggctttacc
ccggcgggttcgccgaaagccctacccaccgcgattgcccgcagagctaacgaacagcatgaggccaaaaagccgtatcaaattcgccttc
tgacgggtgcgtcaatcagcgccgccgctgacgatgtactttctgacgccgatgctgtttcctggcgtgcgccatatcaaacatcgtccggttta
cgtaaaaagatcaatcagggcgcggtgagtttcgttgacctgcatttgagcgaagtggcgcaaatggtcaattacggtttcttcggcgacattg
atgttgccgtcattgaagcatcggcactggcaccggatggtcgagtctggttaaccagcgggatcggtaatgcgccgacctggctgctgcgg
gcgaagaaagtgatcattgaactcaatcactatcacgatccgcgcgttgcagaactggcggatattgtgattcctggcgcgccaccgcggcg
caatagcgtgtcgatcttccatgcaatggatcgcgtcggtacccgctatgtgcaaatcgatccgaaaaagattgtcgccgtcgtggaaaccaac
ttgcccgacgccggtaatatgctggataagcaaaatcccatgtgccagcagattgccgataacgtggtcacgttcttattgcaggaaatggcgc
atgggcgtattccgccggaatttctgccgctgcaaagtggcgtgggcaatatcaataatgcggtaatggcgcgtctgggggaaaacccggta
attcctccgtttatgatgtattcggaagtgctacaggaatcggtggtgcatttactggaaaccggcaaaatcagcggggccagcgcctccagcc
tgacaatctcggccgattccctgcgcaagatttacgacaatatggattactttgccagccgcattgtgttgcgtccgcaggagatttccaataacc
cggaaatcatccgtcgtctgggcgtcatcgctctgaacgtcggcctggagtttgatatttacgggcatgccaactcaacacacgtagccgggg
tcgatctgatgaacggcatcggcggcagcggtgattttgaacgcaacgcgtatctgtcgatctttatggccccgtcgattgctaaagaaggcaa
gatctcaaccgtcgtgccaatgtgcagccatgttgatcacagcgaacacagcgtcaaagtgatcatcaccgaacaagggatcgccgatctgc
gcggtctttccccgcttcaacgcgcccgcactatcattgataattgtgcacatcctatgtatcgggattatctgcatcgctatctggaaaatgcgcc
tggcggacatattcaccacgatcttagccacgtcttcgacttacaccgtaatttaattgcaaccggctcgatgctgggttaatgactcgtcatctcc
aggcatcaaataaaacgaaaggctcagtcgaaagactgggcctttcgttttatctgttgtttgtcggtgaacgctctctactagagtcacactggc
tcaccttcgggtgggcctttctgcgtttatacctagggcgttcggctgcggtccgctcatgaattaattcttagaaaaactcatcgagcatcaaatg
aaactgcaatttattcatatcaggattatcaataccatatttttgaaaaagccgtttctgtaatgaaggagaaaactcaccgaggcagttccatagg
atggcaagatcctggtatcggtctgcgattccgactcgtccaacatcaatacaacctattaatttcccctcgtcaaaaataaggttatcaagtgag
aaatcaccatgagtgacgactgaatccggtgagaatggcaaaagtttatgcatttctttccagacttgttcaacaggccagccattacgctcgtc
atcaaaatcactcgcatcaaccaaaccgttattcattcgtgattgcgcctgagcgagacgaaatacgcgatcgctgttaaaaggacaattacaa
acaggaatcgaatgcaaccggcgcaggaacactgccagcgcatcaacaatattttcacctgaatcaggatattcttctaatacctggaatgctg
ttttcccggggatcgcagtggtgagtaaccatgcatcatcaggagtacggataaaatgcttgatggtcggaagaggcataaattccgtcagcc
agtttagtctgaccatctcatctgtaacatcattggcaacgctacctttgccatgtttcagaaacaactctggcgcatcgggcttcccatacaatcg
atagattgtcgcacctgattgcccgacattatcgcgagcccatttatacccatataaatcagcatccatgttggaatttaatcgcggcctagagca
agacgtttcccgttgaatatggctcataacaccccttgtattactgtttatgtaagcagacagttttattgttcatgaccaaaatcccttaacgtgagtt
ttcgttccactgagcgtcagaccccgtagaaaagatcaaaggatcttcttgagatcctttttttctgcgcgtaatctgctgcttgcaaacaaaaaaa
ccaccgctaccagcggtggtttgtttgccggatcaagagctaccaactctttttccgaaggtaactggcttcagcagagcgcagataccaaata
ctgtccttctagtgtagccgtagttaggccaccacttcaagaactctgtagcaccgcctacatacctcgctctgctaatcctgttaccagtggctg
ctgccagtggcgataagtcgtgtcttaccgggttggactcaagacgatagttaccggataaggcgcagcggtcgggctgaacggggggttc

gtgcacacagcccagcttggagcgaacgacctacaccgaactgagatacctacagcgtgagctatgagaaagcgccacgcttcccgaagg
gagaaaggcggacaggtatccggtaagcggcagggtcggaacaggagagcgcacgagggagcttccagggggaaacgcctggtatcttt
atagtcctgtcgggtttcgccacctctgacttgagcgtcgatttttgtgatgctcgtcaggggggcggagcctatggaaaaacgccagcaacgc
ggcctttttacggttcctggccttttgctggcct

Ptrc-Sbm

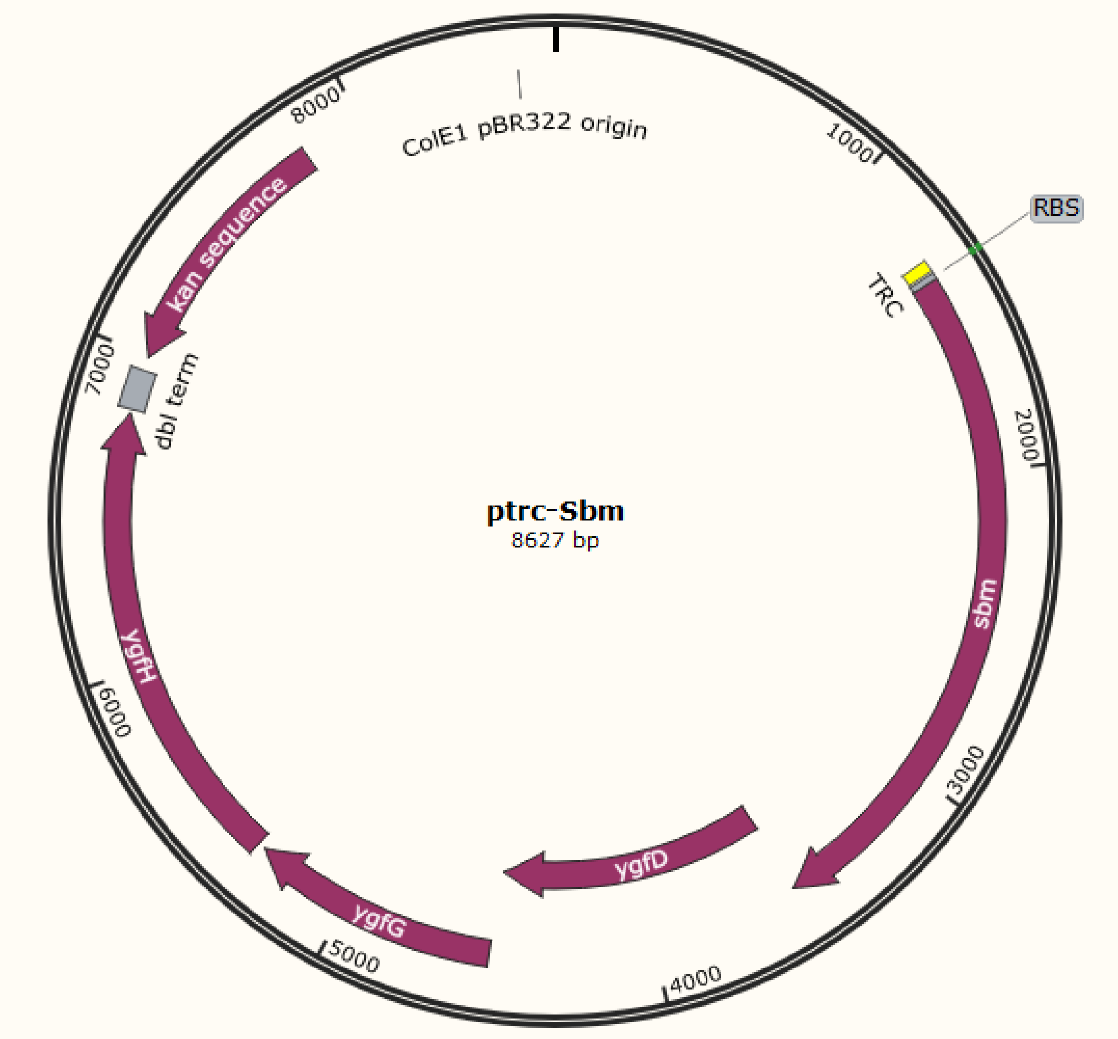

Ptrc-Sbm
>
tgagtgagctgataccgctcgccgcagccgaacgaccgagcgcagcgagtcagtgagcgaggaagcggaagagcgcctgatgcggtatt
ttctccttacgcatctgtgcggtatttcacaccgcatatatggtgcactctcagtacaatctgctctgatgccgcatagttaagccagtatacactcc
gctatcgctacgtgactgggtcatggctgcgccccgacacccgccaacacccgctgacgcgccctgacgggcttgtctgctcccggcatcc
gcttacagacaagctgtgaccgtctccgggagctgcatgtgtcagaggttttcaccgtcatcaccgaaacgcgcgaggcagctgcggtaaag
ctcatcagcgtggtcgtgaagcgattcacagatgtctgcctgttcatccgcgtccagctcgttgagtttctccagaagcgttaatgtctggcttctg
ataaagcgggccatgttaagggcggtttttcctgtttggtcactgatgcctccgtgtaagggggatttctgttcatgggggtaatgataccgatg
aaacgagagaggatgctcacgatacgggttactgatgatgaacatgcccggttactggaacgttgtgagggtaaacaactggcggtatggat
gcggcgggaccagagaaaaatcactcagggtcaatgccagcgcttcgttaatacagatgtaggtgttccacagggtagccagcagcatcctg

cgatgcagatccggaacataatggtgcagggcgctgacttccgcgtttccagactttacgaaacacggaaaccgaagaccattcatgttgttg
ctcaggtcgcagacgttttgcagcagcagtcgcttcacgttcgctcgcgtatcggtgattcattctgctaaccagtaaggcaaccccgccagcc
tagccgggtcctcaacgacaggagcacgatcatgcgcacccgtggggccgccatgccggcgataatggcctgcttctcgccgaaacgtttg
gtggcgggaccagtgacgaaggcttgagcgagggcgtgcaagattccgaataccgcaagcgacaggccgatcatcgtcgcgctccagcg
aaagcggtcctcgccgaaaatgacccagagcgctgccggcacctgtcctacgagttgcatgataaagaagacagtcataagtgcggcgacg
atagtcatgccccgcgcccaccggaaggagctgactgggttgaaggctctcaagggcatcggtcgagatcccggtgctccttggcggcaaa
cttacattaattgcgttttgacaattaatcatcggctcgtataatgtgtggaattgtgattgaattcaaaggaggccatcctaatgtctaacgtgcag
gagtggcaacagcttgccaacaaggaattgagccgtcgggagaaaactgtcgactcgctggttcatcaaaccgcggaagggatcgccatca
agccgctgtataccgaagccgatctcgataatctggaggtgacaggtacccttcctggtttgccgccctacgttcgtggcccgcgtgccactat
gtataccgcccaaccgtggaccatccgtcagtatgctggtttttcaacagcaaaagagtccaacgctttttatcgccgtaacctggccgccggg
caaaaaggtctttccgttgcgtttgaccttgccacccaccgtggctacgactccgataacccgcgcgtggcgggcgacgtcggcaaagcgg
gcgtcgctatcgacaccgtggaagatatgaaagtcctgttcgaccagatcccgctggataaaatgtcggtttcgatgaccatgaatggcgcagt
gctaccagtactggcgttttatatcgtcgccgcagaagagcaaggtgttacacctgataaactgaccggcaccattcaaaacgatattctcaaa
gagtacctctgccgcaacacctatatttacccaccaaaaccgtcaatgcgcattatcgccgacatcatcgcctggtgttccggcaacatgccgc
gatttaataccatcagtatcagcggttaccacatgggtgaagcgggtgccaactgcgtgcagcaggtagcatttacgctcgctgatgggattga
gtacatcaaagcagcaatctctgccggactgaaaattgatgacttcgctcctcgcctgtcgttcttcttcggcatcggcatggatctgtttatgaac
gtcgccatgttgcgtgcggcacgttatttatggagcgaagcggtcagtggatttggcgcacaggacccgaaatcactggcgctgcgtaccca
ctgccagacctcaggctggagcctgactgaacaggatccgtataacaacgttatccgcaccaccattgaagcgctggctgcgacgctgggc
ggtactcagtcactgcataccaacgcctttgacgaagcgcttggtttgcctaccgatttctcagcacgcattgcccgcaacacccagatcatcat
ccaggaagaatcagaactctgccgcaccgtcgatccactggccggatcctattacattgagtcgctgaccgatcaaatcgtcaaacaagcca
gagctattatccaacagatcgacgaagccggtggcatggcgaaagcgatcgaagcaggtctgccaaaacgaatgatcgaagaggcctcag
cgcgcgaacagtcgctgatcgaccagggcaagcgtgtcatcgttggtgtcaacaagtacaaactggatcacgaagacgaaaccgatgtactt
gagatcgacaacgtgatggtgcgtaacgagcaaattgcttcgctggaacgcattcgcgccacccgtgatgatgccgccgtaaccgccgcgtt
gaacgccctgactcacgccgcacagcataacgaaaacctgctggctgccgctgttaatgccgctcgcgttcgcgccaccctgggtgaaattt
ccgatgcgctggaagtcgctttcgaccgttatctggtgccaagccagtgtgttaccggcgtgattgcgcaaagctatcatcagtctgagaaatc
ggcctccgagttcgatgccattgttgcgcaaacggagcagttccttgccgacaatggtcgtcgcccgcgcattctgatcgctaagatgggcca
ggatggacacgatcgcggcgcgaaagtgatcgccagcgcctattccgatctcggtttcgacgtagatttaagcccgatgttctctacacctgaa
gagatcgcccgcctggccgtagaaaacgacgttcacgtagtgggcgcatcctcactggctgccggtcataaaacgctgatcccggaactggt
cgaagcgctgaaaaaatggggacgcgaagatatctgcgtggtcgcgggtggcgtcattccgccgcaggattacgccttcctgcaagagcgc
ggcgtggcggcgatttatggtccaggtacacctatgctcgacagtgtgcgcgacgtactgaatctgataagccagcatcatgattaatgaagc
cacgctggcagaaagtattcgccgcttacgtcagggtgagcgtgccacactcgcccaggccatgacgctggtggaaagccgtcacccgcgt
catcaggcactaagtacgcagctgcttgatgccattatgccgtactgcggtaacaccctgcgactgggcgttaccggcacccccggcgcggg
gaaaagtacctttcttgaggcctttggcatgttgttgattcgagagggattaaaggtcgcggttattgcggtcgatcccagcagcccggtcactg
gcggtagcattctcggggataaaacccgcatgaatgacctggcgcgtgccgaagcggcgtttattcgcccggtaccatcctccggtcatctgg
gcggtgccagtcagcgagcgcgggaattaatgctgttatgcgaagcagcgggttatgacgtagtgattgtcgaaacggttggcgtcgggcag
tcggaaacagaagtcgcccgcatggtggactgttttatctcgttgcaaattgccggtggcggcgatgatctgcagggcattaaaaaagggctg
atggaagtggctgatctgatcgttatcaacaaagacgatggcgataaccataccaatgtcgccattgcccggcatatgtacgagagtgccctg
catattctgcgacgtaaatacgacgaatggcagccacgggttctgacttgtagcgcactggaaaaacgtggaatcgatgagatctggcacgc
catcatcgacttcaaaaccgcgctaactgccagtggtcgtttacaacaagtgcggcaacaacaatcggtggaatggctgcgtaagcagaccg
aagaagaagtactgaatcacctgttcgcgaatgaagatttcgatcgctattaccgccagacgcttttagcggtcaaaaacaatacgctctcacc
gcgcaccggcctgcggcagctcagtgaatttatccagacgcaatattttgattaaaggaatttttatgtcttatcagtatgttaacgttgtcactatc
aacaaagtggcggtcattgagtttaactatggccgaaaacttaatgccttaagtaaagtctttattgatgatcttatgcaggcgttaagcgatctca
accggccggaaattcgctgtatcattttgcgcgcaccgagtggatccaaagtcttctccgcaggtcacgatattcacgaactgccgtctggcgg
tcgcgatccgctctcctatgatgatccattgcgtcaaatcacccgcatgatccaaaaattcccgaaaccgatcatttcgatggtggaaggtagtg

tttggggtggcgcatttgaaatgatcatgagttccgatctgatcatcgccgccagtacctcaaccttctcaatgacgcctgtaaacctcggcgtcc
cgtataacctggtcggcattcacaacctgacccgcgacgcgggcttccacattgtcaaagagctgatttttaccgcttcgccaatcaccgccca
gcgcgcgctggctgtcggcatcctcaaccatgttgtggaagtggaagaactggaagatttcaccttacaaatggcgcaccacatctctgagaa
agcgccgttagccattgccgttatcaaagaagagctgcgtgtactgggcgaagcacacaccatgaactccgatgaatttgaacgtattcaggg
gatgcgccgcgcggtgtatgacagcgaagattaccaggaagggatgaacgctttcctcgaaaaacgtaaacctaatttcgttggtcattaatcc
ctgcgaacgaaggagtaaaaatggaaactcagtggacaaggatgaccgccaatgaagcggcagaaattatccagcataacgacatggtgg
catttagcggctttaccccggcgggttcgccgaaagccctacccaccgcgattgcccgcagagctaacgaacagcatgaggccaaaaagcc
gtatcaaattcgccttctgacgggtgcgtcaatcagcgccgccgctgacgatgtactttctgacgccgatgctgtttcctggcgtgcgccatatc
aaacatcgtccggtttacgtaaaaagatcaatcagggcgcggtgagtttcgttgacctgcatttgagcgaagtggcgcaaatggtcaattacgg
tttcttcggcgacattgatgttgccgtcattgaagcatcggcactggcaccggatggtcgagtctggttaaccagcgggatcggtaatgcgccg
acctggctgctgcgggcgaagaaagtgatcattgaactcaatcactatcacgatccgcgcgttgcagaactggcggatattgtgattcctggc
gcgccaccgcggcgcaatagcgtgtcgatcttccatgcaatggatcgcgtcggtacccgctatgtgcaaatcgatccgaaaaagattgtcgcc
gtcgtggaaaccaacttgcccgacgccggtaatatgctggataagcaaaatcccatgtgccagcagattgccgataacgtggtcacgttcttat
tgcaggaaatggcgcatgggcgtattccgccggaatttctgccgctgcaaagtggcgtgggcaatatcaataatgcggtaatggcgcgtctg
ggggaaaacccggtaattcctccgtttatgatgtattcggaagtgctacaggaatcggtggtgcatttactggaaaccggcaaaatcagcggg
gccagcgcctccagcctgacaatctcggccgattccctgcgcaagatttacgacaatatggattactttgccagccgcattgtgttgcgtccgc
aggagatttccaataacccggaaatcatccgtcgtctgggcgtcatcgctctgaacgtcggcctggagtttgatatttacgggcatgccaactca
acacacgtagccggggtcgatctgatgaacggcatcggcggcagcggtgattttgaacgcaacgcgtatctgtcgatctttatggccccgtcg
attgctaaagaaggcaagatctcaaccgtcgtgccaatgtgcagccatgttgatcacagcgaacacagcgtcaaagtgatcatcaccgaaca
agggatcgccgatctgcgcggtctttccccgcttcaacgcgcccgcactatcattgataattgtgcacatcctatgtatcgggattatctgcatcg
ctatctggaaaatgcgcctggcggacatattcaccacgatcttagccacgtcttcgacttacaccgtaatttaattgcaaccggctcgatgctgg
gttaatgactcgtcatctccaggcatcaaataaaacgaaaggctcagtcgaaagactgggcctttcgttttatctgttgtttgtcggtgaacgctct
ctactagagtcacactggctcaccttcgggtgggcctttctgcgtttatacctagggcgttcggctgcggtccgctcatgaattaattcttagaaa
aactcatcgagcatcaaatgaaactgcaatttattcatatcaggattatcaataccatatttttgaaaaagccgtttctgtaatgaaggagaaaactc
accgaggcagttccataggatggcaagatcctggtatcggtctgcgattccgactcgtccaacatcaatacaacctattaatttcccctcgtcaa
aaataaggttatcaagtgagaaatcaccatgagtgacgactgaatccggtgagaatggcaaaagtttatgcatttctttccagacttgttcaacag
gccagccattacgctcgtcatcaaaatcactcgcatcaaccaaaccgttattcattcgtgattgcgcctgagcgagacgaaatacgcgatcgct
gttaaaaggacaattacaaacaggaatcgaatgcaaccggcgcaggaacactgccagcgcatcaacaatattttcacctgaatcaggatattc
ttctaatacctggaatgctgttttcccggggatcgcagtggtgagtaaccatgcatcatcaggagtacggataaaatgcttgatggtcggaaga
ggcataaattccgtcagccagtttagtctgaccatctcatctgtaacatcattggcaacgctacctttgccatgtttcagaaacaactctggcgcat
cgggcttcccatacaatcgatagattgtcgcacctgattgcccgacattatcgcgagcccatttatacccatataaatcagcatccatgttggaatt
taatcgcggcctagagcaagacgtttcccgttgaatatggctcataacaccccttgtattactgtttatgtaagcagacagttttattgttcatgacc
aaaatcccttaacgtgagttttcgttccactgagcgtcagaccccgtagaaaagatcaaaggatcttcttgagatcctttttttctgcgcgtaatctg
ctgcttgcaaacaaaaaaaccaccgctaccagcggtggtttgtttgccggatcaagagctaccaactctttttccgaaggtaactggcttcagca
gagcgcagataccaaatactgtccttctagtgtagccgtagttaggccaccacttcaagaactctgtagcaccgcctacatacctcgctctgcta
atcctgttaccagtggctgctgccagtggcgataagtcgtgtcttaccgggttggactcaagacgatagttaccggataaggcgcagcggtcg
ggctgaacggggggttcgtgcacacagcccagcttggagcgaacgacctacaccgaactgagatacctacagcgtgagctatgagaaagc
gccacgcttcccgaagggagaaaggcggacaggtatccggtaagcggcagggtcggaacaggagagcgcacgagggagcttccaggg
ggaaacgcctggtatctttatagtcctgtcgggtttcgccacctctgacttgagcgtcgatttttgtgatgctcgtcaggggggcggagcctatgg
aaaaacgccagcaacgcggcctttttacggttcctggccttttgctggccttttgctcacatgttctttcctgcgttatcccctgattctgtggataac
cgtattaccgcctt

pbba-Sbm

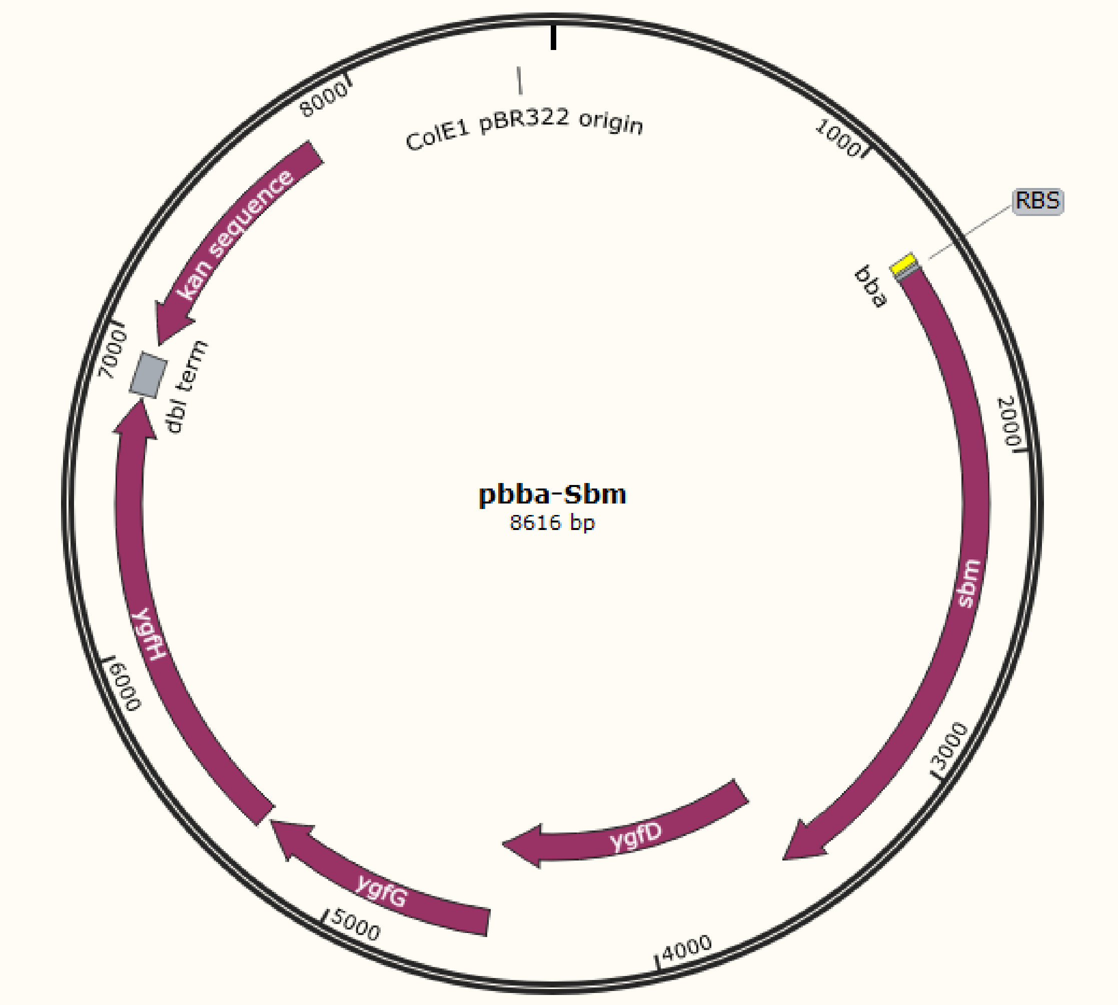

pbba-Sbm

>cgcctttgagtgagctgataccgctcgccgcagccgaacgaccgagcgcagcgagtcagtgagcgaggaagcggaagagcgcctgat
gcggtattttctccttacgcatctgtgcggtatttcacaccgcatatatggtgcactctcagtacaatctgctctgatgccgcatagttaagccagta
tacactccgctatcgctacgtgactgggtcatggctgcgccccgacacccgccaacacccgctgacgcgccctgacgggcttgtctgctccc
ggcatccgcttacagacaagctgtgaccgtctccgggagctgcatgtgtcagaggttttcaccgtcatcaccgaaacgcgcgaggcagctgc
ggtaaagctcatcagcgtggtcgtgaagcgattcacagatgtctgcctgttcatccgcgtccagctcgttgagtttctccagaagcgttaatgtct
ggcttctgataaagcgggccatgttaagggcggttttttcctgtttggtcactgatgcctccgtgtaagggggatttctgttcatgggggtaatgat
accgatgaaacgagagaggatgctcacgatacgggttactgatgatgaacatgcccggttactggaacgttgtgagggtaaacaactggcgg
tatggatgcggcgggaccagagaaaaatcactcagggtcaatgccagcgcttcgttaatacagatgtaggtgttccacagggtagccagcag
catcctgcgatgcagatccggaacataatggtgcagggcgctgacttccgcgtttccagactttacgaaacacggaaaccgaagaccattcat
gttgttgctcaggtcgcagacgttttgcagcagcagtcgcttcacgttcgctcgcgtatcggtgattcattctgctaaccagtaaggcaaccccg
ccagcctagccgggtcctcaacgacaggagcacgatcatgcgcacccgtggggccgccatgccggcgataatggcctgcttctcgccgaa
acgtttggtggcgggaccagtgacgaaggcttgagcgagggcgtgcaagattccgaataccgcaagcgacaggccgatcatcgtcgcgct
ccagcgaaagcggtcctcgccgaaaatgacccagagcgctgccggcacctgtcctacgagttgcatgataaagaagacagtcataagtgcg
gcgacgatagtcatgccccgcgcccaccggaaggagctgactgggttgaaggctctcaagggcatcggtcgagatcccggtgctccttggc
ggcaaacttacattaattgcgttagctagctcagtcctaggtattatgctagcattgaattcaaaggaggccatcctaatgtctaacgtgcaggag

tggcaacagcttgccaacaaggaattgagccgtcgggagaaaactgtcgactcgctggttcatcaaaccgcggaagggatcgccatcaagc
cgctgtataccgaagccgatctcgataatctggaggtgacaggtacccttcctggtttgccgccctacgttcgtggcccgcgtgccactatgtat
accgcccaaccgtggaccatccgtcagtatgctggtttttcaacagcaaaagagtccaacgctttttatcgccgtaacctggccgccgggcaa
aaaggtctttccgttgcgtttgaccttgccacccaccgtggctacgactccgataacccgcgcgtggcgggcgacgtcggcaaagcgggcgt
cgctatcgacaccgtggaagatatgaaagtcctgttcgaccagatcccgctggataaaatgtcggtttcgatgaccatgaatggcgcagtgcta
ccagtactggcgttttatatcgtcgccgcagaagagcaaggtgttacacctgataaactgaccggcaccattcaaaacgatattctcaaagagt
acctctgccgcaacacctatatttacccaccaaaaccgtcaatgcgcattatcgccgacatcatcgcctggtgttccggcaacatgccgcgattt
aataccatcagtatcagcggttaccacatgggtgaagcgggtgccaactgcgtgcagcaggtagcatttacgctcgctgatgggattgagtac
atcaaagcagcaatctctgccggactgaaaattgatgacttcgctcctcgcctgtcgttcttcttcggcatcggcatggatctgtttatgaacgtcg
ccatgttgcgtgcggcacgttatttatggagcgaagcggtcagtggatttggcgcacaggacccgaaatcactggcgctgcgtacccactgc
cagacctcaggctggagcctgactgaacaggatccgtataacaacgttatccgcaccaccattgaagcgctggctgcgacgctgggcggta
ctcagtcactgcataccaacgcctttgacgaagcgcttggtttgcctaccgatttctcagcacgcattgcccgcaacacccagatcatcatccag
gaagaatcagaactctgccgcaccgtcgatccactggccggatcctattacattgagtcgctgaccgatcaaatcgtcaaacaagccagagct
attatccaacagatcgacgaagccggtggcatggcgaaagcgatcgaagcaggtctgccaaaacgaatgatcgaagaggcctcagcgcgc
gaacagtcgctgatcgaccagggcaagcgtgtcatcgttggtgtcaacaagtacaaactggatcacgaagacgaaaccgatgtacttgagat
cgacaacgtgatggtgcgtaacgagcaaattgcttcgctggaacgcattcgcgccacccgtgatgatgccgccgtaaccgccgcgttgaacg
ccctgactcacgccgcacagcataacgaaaacctgctggctgccgctgttaatgccgctcgcgttcgcgccaccctgggtgaaatttccgatg
cgctggaagtcgctttcgaccgttatctggtgccaagccagtgtgttaccggcgtgattgcgcaaagctatcatcagtctgagaaatcggcctc
cgagttcgatgccattgttgcgcaaacggagcagttccttgccgacaatggtcgtcgcccgcgcattctgatcgctaagatgggccaggatgg
acacgatcgcggcgcgaaagtgatcgccagcgcctattccgatctcggtttcgacgtagatttaagcccgatgttctctacacctgaagagatc
gcccgcctggccgtagaaaacgacgttcacgtagtgggcgcatcctcactggctgccggtcataaaacgctgatcccggaactggtcgaag
cgctgaaaaaatggggacgcgaagatatctgcgtggtcgcgggtggcgtcattccgccgcaggattacgccttcctgcaagagcgcggcgt
ggcggcgatttatggtccaggtacacctatgctcgacagtgtgcgcgacgtactgaatctgataagccagcatcatgattaatgaagccacgct
ggcagaaagtattcgccgcttacgtcagggtgagcgtgccacactcgcccaggccatgacgctggtggaaagccgtcacccgcgtcatcag
gcactaagtacgcagctgcttgatgccattatgccgtactgcggtaacaccctgcgactgggcgttaccggcacccccggcgcggggaaaa
gtacctttcttgaggcctttggcatgttgttgattcgagagggattaaaggtcgcggttattgcggtcgatcccagcagcccggtcactggcggt
agcattctcggggataaaacccgcatgaatgacctggcgcgtgccgaagcggcgtttattcgcccggtaccatcctccggtcatctgggcggt
gccagtcagcgagcgcgggaattaatgctgttatgcgaagcagcgggttatgacgtagtgattgtcgaaacggttggcgtcgggcagtcgga
aacagaagtcgcccgcatggtggactgttttatctcgttgcaaattgccggtggcggcgatgatctgcagggcattaaaaaagggctgatgga
agtggctgatctgatcgttatcaacaaagacgatggcgataaccataccaatgtcgccattgcccggcatatgtacgagagtgccctgcatatt
ctgcgacgtaaatacgacgaatggcagccacgggttctgacttgtagcgcactggaaaaacgtggaatcgatgagatctggcacgccatcat
cgacttcaaaaccgcgctaactgccagtggtcgtttacaacaagtgcggcaacaacaatcggtggaatggctgcgtaagcagaccgaagaa
gaagtactgaatcacctgttcgcgaatgaagatttcgatcgctattaccgccagacgcttttagcggtcaaaaacaatacgctctcaccgcgca
ccggcctgcggcagctcagtgaatttatccagacgcaatattttgattaaaggaatttttatgtcttatcagtatgttaacgttgtcactatcaacaaa
gtggcggtcattgagtttaactatggccgaaaacttaatgccttaagtaaagtctttattgatgatcttatgcaggcgttaagcgatctcaaccggc
cggaaattcgctgtatcattttgcgcgcaccgagtggatccaaagtcttctccgcaggtcacgatattcacgaactgccgtctggcggtcgcga
tccgctctcctatgatgatccattgcgtcaaatcacccgcatgatccaaaaattcccgaaaccgatcatttcgatggtggaaggtagtgtttgggg
tggcgcatttgaaatgatcatgagttccgatctgatcatcgccgccagtacctcaaccttctcaatgacgcctgtaaacctcggcgtcccgtataa
cctggtcggcattcacaacctgacccgcgacgcgggcttccacattgtcaaagagctgatttttaccgcttcgccaatcaccgcccagcgcgc
gctggctgtcggcatcctcaaccatgttgtggaagtggaagaactggaagatttcaccttacaaatggcgcaccacatctctgagaaagcgcc
gttagccattgccgttatcaaagaagagctgcgtgtactgggcgaagcacacaccatgaactccgatgaatttgaacgtattcaggggatgcg
ccgcgcggtgtatgacagcgaagattaccaggaagggatgaacgctttcctcgaaaaacgtaaacctaatttcgttggtcattaatccctgcga
acgaaggagtaaaaatggaaactcagtggacaaggatgaccgccaatgaagcggcagaaattatccagcataacgacatggtggcatttag
cggctttaccccggcgggttcgccgaaagccctacccaccgcgattgcccgcagagctaacgaacagcatgaggccaaaaagccgtatca

aattcgccttctgacgggtgcgtcaatcagcgccgccgctgacgatgtactttctgacgccgatgctgtttcctggcgtgcgccatatcaaacat
cgtccggtttacgtaaaaagatcaatcagggcgcggtgagtttcgttgacctgcatttgagcgaagtggcgcaaatggtcaattacggtttcttc
ggcgacattgatgttgccgtcattgaagcatcggcactggcaccggatggtcgagtctggttaaccagcgggatcggtaatgcgccgacctg
gctgctgcgggcgaagaaagtgatcattgaactcaatcactatcacgatccgcgcgttgcagaactggcggatattgtgattcctggcgcgcc
accgcggcgcaatagcgtgtcgatcttccatgcaatggatcgcgtcggtacccgctatgtgcaaatcgatccgaaaaagattgtcgccgtcgt
ggaaaccaacttgcccgacgccggtaatatgctggataagcaaaatcccatgtgccagcagattgccgataacgtggtcacgttcttattgcag
gaaatggcgcatgggcgtattccgccggaatttctgccgctgcaaagtggcgtgggcaatatcaataatgcggtaatggcgcgtctgggga
aaacccggtaattcctccgtttatgatgtattcggaagtgctacaggaatcggtggtgcatttactggaaaccggcaaaatcagcggggccagc
gcctccagcctgacaatctcggccgattccctgcgcaagatttacgacaatatggattactttgccagccgcattgtgttgcgtccgcaggagat
ttccaataacccggaaatcatccgtcgtctgggcgtcatcgctctgaacgtcggcctggagtttgatatttacgggcatgccaactcaacacac
gtagccggggtcgatctgatgaacggcatcggcggcagcggtgattttgaacgcaacgcgtatctgtcgatctttatggccccgtcgattgcta
aagaaggcaagatctcaaccgtcgtgccaatgtgcagccatgttgatcacagcgaacacagcgtcaaagtgatcatcaccgaacaagggat
cgccgatctgcgcggtctttccccgcttcaacgcgcccgcactatcattgataattgtgcacatcctatgtatcgggattatctgcatcgctatctg
gaaaatgcgcctggcggacatattcaccacgatcttagccacgtcttcgacttacaccgtaatttaattgcaaccggctcgatgctgggttaatg
actcgtcatctccaggcatcaaataaaacgaaaggctcagtcgaaagactgggcctttcgttttatctgttgtttgtcggtgaacgctctctactag
agtcacactggctcaccttcgggtgggcctttctgcgtttatacctagggcgttcggctgcggtccgctcatgaattaattcttagaaaaactcatc
gagcatcaaatgaaactgcaatttattcatatcaggattatcaataccatatttttgaaaaagccgtttctgtaatgaaggagaaaactcaccgagg
cagttccataggatggcaagatcctggtatcggtctgcgattccgactcgtccaacatcaatacaacctattaatttcccctcgtcaaaaataagg
ttatcaagtgagaaatcaccatgagtgacgactgaatccggtgagaatggcaaaagtttatgcatttctttccagacttgttcaacaggccagcc
attacgctcgtcatcaaaatcactcgcatcaaccaaaccgttattcattcgtgattgcgcctgagcgagacgaaatacgcgatcgctgttaaaag
gacaattacaaacaggaatcgaatgcaaccggcgcaggaacactgccagcgcatcaacaatattttcacctgaatcaggatattcttctaatac
ctggaatgctgttttcccggggatcgcagtggtgagtaaccatgcatcatcaggagtacggataaaatgcttgatggtcggaagaggcataaat
tccgtcagccagtttagtctgaccatctcatctgtaacatcattggcaacgctacctttgccatgtttcagaaacaactctggcgcatcgggcttcc
catacaatcgatagattgtcgcacctgattgcccgacattatcgcgagcccatttatacccatataaatcagcatccatgttggaatttaatcgcgg
cctagagcaagacgtttcccgttgaatatggctcataacaccccttgtattactgtttatgtaagcagacagttttattgttcatgaccaaaatccctt
aacgtgagttttcgttccactgagcgtcagaccccgtagaaaagatcaaaggatcttcttgagatcctttttttctgcgcgtaatctgctgcttgcaa
acaaaaaaaccaccgctaccagcggtggtttgtttgccggatcaagagctaccaactctttttccgaaggtaactggcttcagcagagcgcag
ataccaaatactgtccttctagtgtagccgtagttaggccaccacttcaagaactctgtagcaccgcctacatacctcgctctgctaatcctgttac
cagtggctgctgccagtggcgataagtcgtgtcttaccgggttggactcaagacgatagttaccggataaggcgcagcggtcgggctgaacg
gggggttcgtgcacacagcccagcttggagcgaacgacctacaccgaactgagatacctacagcgtgagctatgagaaagcgccacgcttc
ccgaagggagaaaggcggacaggtatccggtaagcggcagggtcggaacaggagagcgcacgagggagcttccagggggaaacgcct
ggtatctttatagtcctgtcgggtttcgccacctctgacttgagcgtcgatttttgtgatgctcgtcaggggggcggagcctatggaaaaacgcc
agcaacgcggcctttttacggttcctggccttttgctggccttttgctcacatgttctttcctgcgttatcccctgattctgtggataaccgtattac

plac-ABb

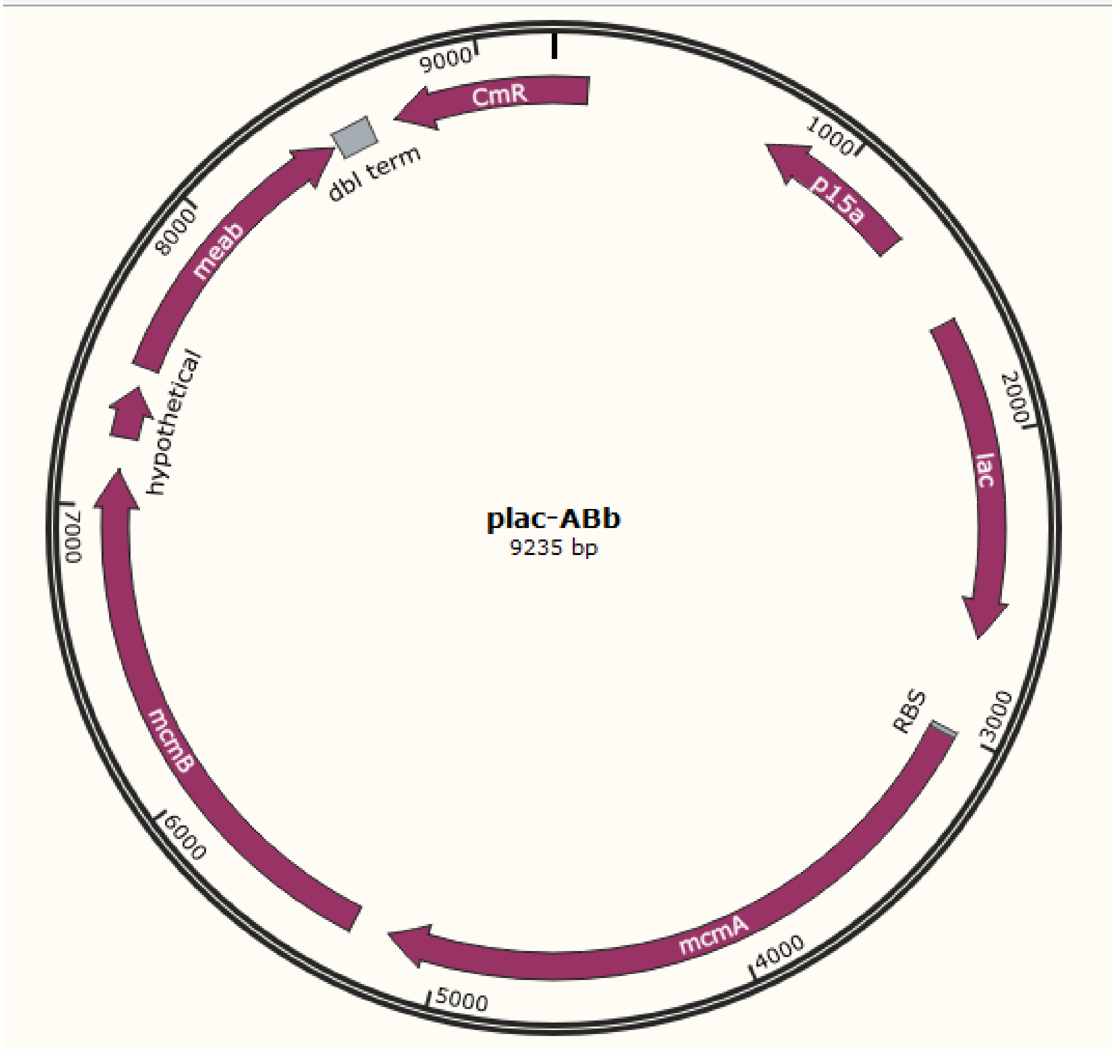

plac-ABb

>gctgaacggtctggttataggtacattgagcaactgactgaaatgcctcaaaatgttctttacgatgccattgggatatatcaacggtggtatat
ccagtgatttttttctccattttagcttccttagctcctgaaaatctcgataactcaaaaaatacgcccggtagtgatcttatttcattatggtgaaagtt
ggaacctcttacgtgccgatcaacgtctcattttcgccaaaagttggcccagggcttcccggtatcaacagggacaccaggatttatttattctgc
gaagtgatcttccgtcacaggtatttattcggcgcaaagtgcgtcgggtgatgctgccaacttactgatttagtgtatgatggtgtttttgaggtgct
ccagtggcttctgtttctatcagctgtccctcctgttcagctactgacggggtggtgcgtaacggcaaaagcaccgccggacatcagcgctagc
ggagtgtatactggcttactatgttggcactgatgagggtgtcagtgaagtgcttcatgtggcaggagaaaaaaggctgcaccggtgcgtcag
cagaatatgtgatacaggatatattccgcttcctcgctcactgactcgctacgctcggtcgttcgactgcggcgagcggaaatggcttacgaac
ggggcggagatttcctggaagatgccaggaagatacttaacagggaagtgagagggccgcggcaaagccgtttttccataggctccgcccc
cctgacaagcatcacgaaatctgacgctcaaatcagtggtggcgaaacccgacaggactataaagataccaggcgtttccccctggcggctc
cctcgtgcgctctcctgttcctgcctttcggtttaccggtgtcattccgctgttatggccgcgtttgtctcattccacgcctgacactcagttccggg
taggcagttcgctccaagctggactgtatgcacgaaccccccgttcagtccgaccgctgcgccttatccggtaactatcgtcttgagtccaacc
cggaaagacatgcaaaagcaccactggcagcagccactggtaattgatttagaggagttagtcttgaagtcatgcgccggttaaggctaaact
gaaaggacaagttttggtgactgcgctcctccaagccagttacctcggttcaaagagttggtagctcagagaaccttcgaaaaaccgccctgc
aaggcggttttttcgttttcagagcaagagattacgcgcagaccaaaacgatctcaagaagatcatcttattaatcagataaaatatttctagatttc
agtgcaatttatctcttcaaatgtagcacctgaagtcagccccatacgatataagttgtaattctcaccgcttacagacaagctgtgaccgtctccg

ggagctgcatgtgtcagaggttttcaccgtcatcaccgaaacgcgcgaggcagcagatcaattcgcgcgcgaaggcgaagcggcatgcatt
tacgttgacaccatcgaatggtgcaaaacctttcgcggtatggcatgatagcgcccggaagagagtcaattcagggtggtgaatgtgaaacca
gtaacgttatacgatgtcgcagagtatgccggtgtctcttatcagaccgtttcccgcgtggtgaaccaggccagccacgtttctgcgaaaacgc
gggaaaaagtggaagcggcgatggcggagctgaattacattcccaaccgcgtggcacaacaactggcgggcaaacagtcgttgctgattg
gcgttgccacctccagtctggccctgcacgcgccgtcgcaaattgtcgcggcgattaaatctcgcgccgatcaactgggtgccagcgtggtg
gtgtcgatggtagaacgaagcggcgtcgaagcctgtaaagcggcggtgcacaatcttctcgcgcaacgcgtcagtgggctgatcattaacta
tccgctggatgaccaggatgccattgctgtggaagctgcctgcactaatgttccggcgttatttcttgatgtctctgaccagacacccatcaaca
gtattattttctcccatgaagacggtacgcgactgggcgtggagcatctggtcgcattgggtcaccagcaaatcgcgctgttagcgggcccatt
aagttctgtctcggcgcgtctgcgtctggctggctggcataaatatctcactcgcaatcaaattcagccgatagcggaacgggaaggcgactg
gagtgccatgtccggttttcaacaaaccatgcaaatgctgaatgagggcatcgttcccactgcgatgctggttgccaacgatcagatggcgctg
ggcgcaatgcgcgccattaccgagtccgggctgcgcgttggtgcggatatctcggtagtgggatacgacgataccgaagacagctcatgtta
tatcccgccgtcaaccaccatcaaacaggattttcgcctgctggggcaaaccagcgtggaccgcttgctgcaactctctcagggccaggcgg
tgaagggcaatcagctgttgcccgtctcactggtgaaaagaaaaaccaccctggcgcccaatacgcaaaccgcctctccccgcgcgttggc
cgattcattaatgcagctggcacgacaggtttcccgactggaaagcgggcagtgagcgcaacgcaattaatgtgagttagcgcgaattgatct
ggtttgacagcttatcatcgactgcacggtgcaccaatgcttctggcgtcaggcagccatcggaagctgtggtatggctgtgcaggtcgtaaat
cactgcataattcgtgtcgctcaaggcgcactcccgttctggataatgttttttgcgccgacatcataacggttctggcaaatattctgaaatgagc
tgttgacaattaatcatccggctcgtataatgtgtggaattgtgagcggataacaatttcacacaggaaacagtgaacgttaaaaaggaatttcgt
cagacgcacgatgagctcccgcatccccgatttcgcctccgtcgcctacgcggccgacggcttcaaggcgccgcccccgccggccgggg
agccgtggatgacgcccgagggcattccggtgaagggcttctacggccccgaggaccgcgagggctgcgaggggatcgactcgttcccc
ggcctgccgccctacctgcgcggcccctatccggcgatgtacgtcacccagccctggacgatccggcaatatgccggcttctcgacggccg
aggattcgaacgccttctatcggcgcaacctcgccgcgggccagaaaggcctctcggtcgccttcgatctcgcgacccaccgcggctacga
ttcggaccacccgcgcgtctcgggtgatgtcggcatggcgggtgtcgcgatcgactcgatctacgacatgcgcacgctgttctccggcattcc
gctcgatgagatgacggtgtcgatgacgatgaacggcgcggtgctgccggtgctcgcgctctacatcgtcgcggccgaggagcagggcgt
gccgcccgaaaagctcgccggcacgatccagaacgacatcctcaaggagttcatggtccgcaacacctacatctatcccccccaagggatcg
atgcggatcatctcggacatcttcggctacacctcgaagaacatgccgaagttcaactcgatctcgatctccggctaccacatgcaggaggcc
ggggcgacgcaggatctggagctcgcctacaccctcgcggacggcgtcgagtacatcaaggcaggcctcgcggcgggcctcaccatcga
ccagttcgcgccgcgcctgtcgttcttctgggcgatcgggatgaacttcttcatggagatcgccaagatgcgggccgcacgcctgatctgggc
caagctcgtcaaagagttcgagccgaagagcgacaagtcgctgccgctgcgcacccattcccagacgtccggctggtcgctgaccgcgca
ggacgtgttcaacaacgtcacccgcacctgcatcgaggcgatggcggcgacgcagggcggcacccagtcgctccacaccaacgcgctcg
acgaggctttggcgctgccgaccgacttctcggcccgcatcgcccgcaacacccagctcttcctgcagcaggagagcggcaccacgcgga
tcatcgatccgtggggcggctcctactatgtcgagcggctgacccgggacatcgccgcccgcgcctgggagcatatccgcgaggtcgagg
cgctcggcggcatggccaaggccatcgaggccggcatcccgaagctgcgcatcgaggaggcggcggcccgcgcccaggcgcggatcg
attccgggcgccagaccatcgtcggcatcaacaagtacaagcccgacgacgagatgaagatcgacctgctgcgcgtcgacaacgccgacg
tgcgcgccaagcagatcgacaagctcaagcgcctgcgcgccgagcgcaaccaggccgacgtcgatgcggctctggccgcgctgacgaa
ggctgcggatggcgagggcaacctgctcgaactggcggtgaacgcggcgcgggccaaagccacggtcggcgagatctcggaggcgctg
gagaaggcctggggccgtcaccgcgcccagatccgctcgatctcgggcgtctacaagcgggaggtgggcggtatgtcgccggtggtgga
gaaggtccgcggcctcgtcgaggccttcgaggagaatgacgggcgccgcccgcgcatcctggtcgccaagatgggccaggacgggcac
gaccgcggccagaaggtgatcgcctcggcgttcgccgatctcggcttcgacgtggatatcgggccgctcttcgccacgccggacgaggcc
gcgcggcaggcggtggagaacgacgtgcacatcgtcggcgtctcctcgctggccgcgggccacctgacgctggtgccggaactcaaggc
cgcgctgaagcaggagggccgcgacgacgtgatgatcgtggtcggcggcgtgatcccgccgggcgactacgacgcgctctacgccgcg
ggcgcctcggcgatcttcccgccgggcaccgtgatcgcggaagcggccgtgaaccttctgggcgaactcaacacgcgcctgggctacggc
gaacggcaggcggccgagtagtccgcttttccagtaaagtcggttgtcgcctgcccgcgccccgtgcgagggcttgcggccaagtcgccgc
tctttgaagaagcggtcgggaaacgccgaatcgtcaaggtctcgcccaagatggaagatcgtccgctcgccgagttgttcgagcccgccacc
cgcgagcgctggcgccaagccatcgaagccgcgctgaagggggcggacttcgagaagcgtctcgtgtcgaagaccgccgacggcctgc

gcatcgagcccttttacgagccggcggcttcggtggcccagccggtgcgggcgcccggcccgtggcgtctggcgcagcgcatcgaccatc
ccgatgccgagaaggccggggcacaggcgctggctgacctcgaaggcggggccgatgcgctggtcctcgtccattgcgacgcccgtgcc
gcccgcggcttcggcctcgaatttccgtccgtcgatgcgctggatgcggcgctgacgggcgtgatgcttccgctcatcgccctgcggctcgat
gccggtccggccggattcgagacggcggcgcttctcaagcaactgatcgagcgccgcggcgacgacccgtccgcgctcgacctcgatctc
ggtctcgacccgatcggcacccgcgccgccaccggccggctcgaccaggggtgggaggcacgcctctccgagatcgtcaccgccttcgc
cggataccgtgggcgcgcggccttggccgacgcccggccctatcacgaggcgggggcgagcgaggtgcaggaactggccgccgtgctc
gcgacggcggtcgcctatctgcgggcgctggaggccggcggccacgatctggcgctcgcgcgcgaccagatcgcggtcctgctggtggc
ggatgccgacgaattcctgaccatcgccaagttccgggcgatccggcggctctgggcccgggtcgagcaggcctgcggcctcgagccga
agccgctgcgggtcctggccgagaccgcgtggcggatgatgagccgccgtgacccattcgtgaacatcctgcgcaccaccatggcctcgg
cctccgccggcctcggcggggcggattcggtgaccgcgctgccctacaccctggcgctcggtctgcccgacgcctttgcccggcgggtgg
tgcgcaacagccagatcgtgctgatcgaggaatcgaacctcgccaaggtctccgatccggcggccggcgcgggcggcttcgaggcgctg
accaccgaactgaccgagaaggcctgggcggccttccaagagatcgagcgagagggcgggatcgccgcgagcctggagtccggcgcct
tcgcgggtcggatcgcggcggtggcgcaggcccgggcacgggcggtggcaacccgcaaggagccgctgaccggcgccagcgagttcc
ccttcctcgccgagaagccggtgacggtgctcgacgtggcgccgaaccccgccgcttcctcagaggggccttgccctcgcaacgcctcg
ccgaaccctacgaggccctgcgcgacgcctccgacgccgtccgcgagcggacgggcaagcgcccggcggtcttcctggccaatctcggt
cccgtggcggttcacaatgcccgctcgaccttcgctgccaacgccttcgctgcgggtggcatcgaggccatcggcaatgacggcttctccga
catgcaggccctggccgatgccttcaaggcgtcgggcgcgcggatcgcctgcctgtgctcgtccgacaccgtctaccagaccgaggccgt
gcccgcggccgaggcactaaaggcggccggcgccggcacgatctacctcgccggcaaacccgccgaggccgaggctctgcgccaagc
gggcgtcgaaggcttcctgttcgccggttgcgacctgttggcgctcctgcgggaagcggccgagcgcgcgaagggctgactaacactcaa
ggccgcgtgagcaaccttgacactgcgggacgcaagacgatcttccggctgaaacgggccgacacggccccatgccctgagccggatgt
catccccatgcgccttccgatccttgccgtcgccacctttgccatcgccgtcggcagcgccggaacatcggaggcggccaagcgcaaggtg
caggtgccgcatcgctacgacggccgctggagcatcgaggtcgtgaccctcgacggcccctgcgaccgggcctatcgctacggcatccag
attgaccgtggcggggcacgattgggattttgggcactcgacgcccgcgcgacgccccggcatgaaggggccatgagcgcgacccttccc
gacatggacacactccgcgagcggcttctggccggcgaccgagcggcgctcgcccgcgccatcacgctggccgagtcgcgccgggccg
atcaccgggcggcagtgcgcgacctcatcgacgcggtgctgccgcagacggggcgggcgatccgcgttggcatcaccggcgtgccggg
cgtcggcaaatccaccaccatcgacgcgctcggctcgctcctgaccgcggcgggtcacaaggtggcggtgctcgccgtcgatccgtcctcg
acccgcaccggcggctcgatcctcggcgacaagacgcggatggcgcggctcgccatcgaccgcaacgccttcatccggccctcgccctcc
tcgggcacgctcggcggcgtcgcggccaagacccgcgagaccatgctgctgtgcgaggcggcgggtttcgacgtgatcctggtggagac
cgtcggcgtcggccagtcggagacggcggtggccgatctcaccgactttttcctcgtgctgatgctgccgggcgccggcgatgagcttcag
ggcatcaagaagggcattctcgaactcgccgacatgattgcggtcaacaaggccgacgatggcgacggcgagcggcgggcgagtgccgc
cgcctccgagtaccgcgctgcgctccacatcctgacgccgccttccgccacctggacgccgccggtggtcacgatctcgggcctgcacggc
aaggggctcgattccctctggagccggatcgaggatcaccgcagcaagctcaccgcgaccggcgagatcgcgggcaagcgccgcgagc
aggacgtgaagtggatgtgggcgctcgttcacgagcggctgcaccagcgcctcgtcggcagcgccgaggtgcggcaggccaccgccga
ggctgagcgggcggttgcgggcggcgagcactcgcccgctgccggcgccgatgcgatcgcgaccctgatcgggttgtgagccgtctgctc
tccaggcatcaaataaaacgaaaggctcagtcgaaagactgggcctttcgttttatctgttgtttgtcggtgaacgctctctactagagtcacact
ggctcaccttcgggtgggcctttctgcgtttatacctagggcgttcggctgcggctggcgctgggcctgtttctggcgctgggcgtttaagggc
accaataactgccttaaaaaaattacgccccgccctgccactcatcgcagtactgttgtaattcattaagcattctgccgacatggaagccatca
cagacggcatgatgaacctgaatcgccagcggcatcagcaccttgtcgccttgcgtataatatttgcccatggtgaaaacgggggcgaagaa
gttgtccatattggccacgtttaaatcaaaactggtgaaactcacccagggattggctgagacgaaaaacatattctcaataaaccctttaggga
aataggccaggttttcaccgtaacacgccacatcttgcgaatatatgtgtagaaactgccggaaatcgtcgtggtattcactccagagcgatga
aaacgtttcagtttgctcatggaaaacggtgtaacaagggtgaacactatcccatatcaccagctcaccgtctttcattgccatacggaattccgg
atgagcattcatcaggcgggcaagaatgtgaataaaggccggataaaacttgtgcttatttttctttacggtctttaaaaaggccgtaatatcca

pbba-ABb

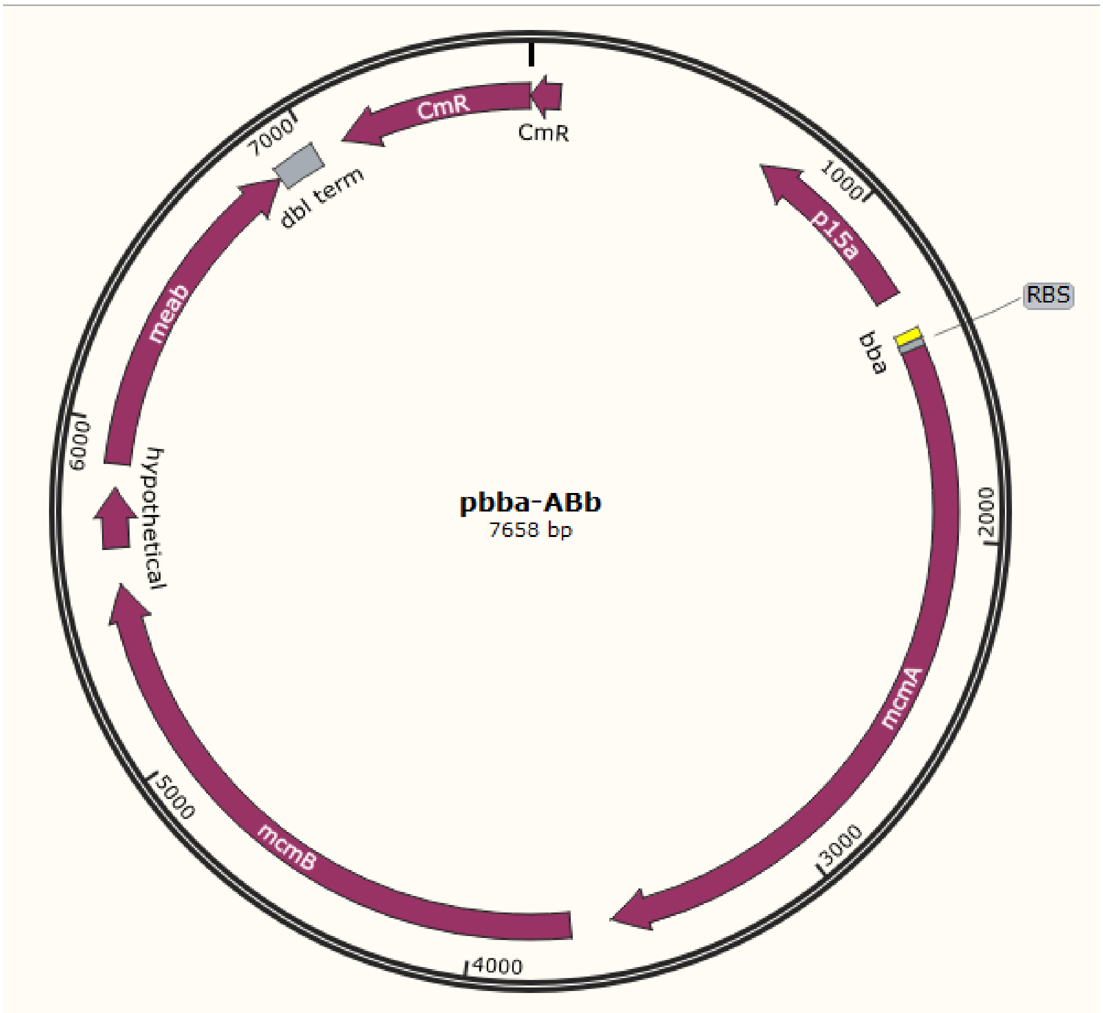

pbba-ABb

>gagcaactgactgaaatgcctcaaaatgttctttacgatgccattgggatatatcaacggtggtatatccagtgatttttttctccattttagcttcct
tagctcctgaaaatctcgataactcaaaaaatacgcccggtagtgatcttatttcattatggtgaaagttggaacctcttacgtgccgatcaacgtc
tcattttcgccaaaagttggcccagggcttcccggtatcaacagggacaccaggatttatttattctgcgaagtgatcttccgtcacaggtatttatt
cggcgcaaagtgcgtcgggtgatgctgccaacttactgatttagtgtatgatggtgtttttgaggtgctccagtggcttctgtttctatcagctgtcc
ctcctgttcagctactgacggggtggtgcgtaacggcaaaagcaccgccggacatcagcgctagcggagtgtatactggcttactatgttggc
actgatgagggtgtcagtgaagtgcttcatgtggcaggagaaaaaaggctgcaccggtgcgtcagcagaatatgtgatacaggatatattccg
cttcctcgctcactgactcgctacgctcggtcgttcgactgcggcgagcggaaatggcttacgaacggggcggagatttcctggaagatgcc
aggaagatacttaacagggaagtgagagggccgcggcaaagccgtttttccataggctccgcccccctgacaagcatcacgaaatctgacg
ctcaaatcagtggtggcgaaacccgacaggactataaagataccaggcgtttccccctggcggctccctcgtgcgctctcctgttcctgccttt
cggtttaccggtgtcattccgctgttatggccgcgtttgtctcattccacgcctgacactcagttccgggtaggcagttcgctccaagctggactg
tatgcacgaaccccccgttcagtccgaccgctgcgccttatccggtaactatcgtcttgagtccaacccggaaagacatgcaaaagcaccact
ggcagcagccactggtaattgatttagaggagttagtcttgaagtcatgcgccggttaaggctaaactgaaaggacaagttttggtgactgcgc
tcctccaagccagttacctcggttcaaagagttggtagctcagagaaccttcgaaaaaccgccctgcaaggcggttttttcgttttcagagcaag
agattacgcgcagaccaaaacgatctcaagaagatcatcttattaatcagataaaatatttctagatttcagtgcaatttatctcttcaaatgtagca
cctgaagtcagccccatacgatataagttgtaattctcacagctagctcagtcctaggtattatgctagcattgaattcaaaggaggccatcctaa
tgagctcccgcatccccgatttcgcctccgtcgcctacgcggccgacggcttcaaggcgccgcccccgccggccggggagccgtggatga

cgcccgagggcattccggtgaagggcttctacggccccgaggaccgcgagggctgcgaggggatcgactcgttccccggcctgccgccc
tacctgcgcggcccctatccggcgatgtacgtcacccagccctggacgatccggcaatatgccggcttctcgacggccgaggattcgaacg
ccttctatcggcgcaacctcgccgcgggccagaaaggcctctcggtcgccttcgatctcgcgacccaccgcggctacgattcggaccaccc
gcgcgtctcgggtgatgtcggcatggcgggtgtcgcgatcgactcgatctacgacatgcgcacgctgttctccggcattccgctcgatgagat
gacggtgtcgatgacgatgaacggcgcggtgctgccggtgctcgcgctctacatcgtcgcggccgaggagcagggcgtgccgcccgaaa
agctcgccggcacgatccagaacgacatcctcaaggagttcatggtccgcaacacctacatctatcccccaagggatcgatgcggatcatc
tcggacatcttcggctacacctcgaagaacatgccgaagttcaactcgatctcgatctccggctaccacatgcaggaggccggggcgacgca
ggatctggagctcgcctacaccctcgcggacggcgtcgagtacatcaaggcaggcctcgcggcgggcctcaccatcgaccagttcgcgcc
gcgcctgtcgttcttctgggcgatcgggatgaacttcttcatggagatcgccaagatgcgggccgcacgcctgatctgggccaagctcgtcaa
agagttcgagccgaagagcgacaagtcgctgccgctgcgcacccattcccagacgtccggctggtcgctgaccgcgcaggacgtgttcaa
caacgtcacccgcacctgcatcgaggcgatggcggcgacgcagggcggcacccagtcgctccacaccaacgcgctcgacgaggctttgg
cgctgccgaccgacttctcggcccgcatcgcccgcaacacccagctcttcctgcagcaggagagcggcaccacgcggatcatcgatccgt
ggggcggctcctactatgtcgagcggctgacccgggacatcgccgcccgcgcctgggagcatatccgcgaggtcgaggcgctcggcggc
atggccaaggccatcgaggccggcatcccgaagctgcgcatcgaggaggcggcggcccgcgcccaggcgcggatcgattccgggcgc
cagaccatcgtcggcatcaacaagtacaagcccgacgacgagatgaagatcgacctgctgcgcgtcgacaacgccgacgtgcgcgccaa
gcagatcgacaagctcaagcgcctgcgcgccgagcgcaaccaggccgacgtcgatgcggctctggccgcgctgacgaaggctgcggat
ggcgagggcaacctgctcgaactggcggtgaacgcggcgcgggccaaagccacggtcggcgagatctcggaggcgctggagaaggcc
tggggccgtcaccgcgcccagatccgctcgatctcgggcgtctacaagcgggaggtgggcggtatgtcgccggtggtggagaaggtccgc
ggcctcgtcgaggccttcgaggagaatgacgggcgccgcccgcgcatcctggtcgccaagatgggccaggacgggcacgaccgcggcc
agaaggtgatcgcctcggcgttcgccgatctcggcttcgacgtggatatcgggccgctcttcgccacgccggacgaggccgcgcggcagg
cggtggagaacgacgtgcacatcgtcggcgtctcctcgctggccgcgggccacctgacgctggtgccggaactcaaggccgcgctgaag
caggagggccgcgacgacgtgatgatcgtggtcggcggcgtgatcccgccgggcgactacgacgcgctctacgccgcgggcgcctcgg
cgatcttcccgccgggcaccgtgatcgcggaagcggccgtgaaccttctgggcgaactcaacacgcgcctgggctacggcgaacggcag
gcggccgagtagtccgcttttccagtaaagtcggttgtcgcctgcccgcgccccgtgcgagggcttgcggccaagtcgccgctctttgaaga
agcggtcgggaaacgccgaatcgtcaaggtctcgcccaagatggaagatcgtccgctcgccgagttgttcgagcccgccacccgcgagcg
ctggcgccaagccatcgaagccgcgctgaagggggcggacttcgagaagcgtctcgtgtcgaagaccgccgacggcctgcgcatcgagc
ccttttacgagccggcggcttcggtggcccagccggtgcgggcgcccggcccgtggcgtctggcgcagcgcatcgaccatcccgatgccg
agaaggccggggcacaggcgctggctgacctcgaaggcggggccgatgcgctggtcctcgtccattgcgacgcccgtgccgcccgcgg
cttcggcctcgaatttccgtccgtcgatgcgctggatgcggcgctgacgggcgtgatgcttccgctcatcgccctgcggctcgatgccggtcc
ggccggattcgagacggcggcgcttctcaagcaactgatcgagcgccgcggcgacgacccgtccgcgctcgacctcgatctcggtctcga
cccgatcggcacccgcgccgccaccggccggctcgaccaggggtgggaggcacgcctctccgagatcgtcaccgccttcgccggatacc
gtgggcgcgcggccttggccgacgcccggccctatcacgaggcgggggcgagcgaggtgcaggaactggccgccgtgctcgcgacgg
cggtcgcctatctgcgggcgctggaggccggcggccacgatctggcgctcgcgcgcgaccagatcgcggtcctgctggtggcggatgcc
gacgaattcctgaccatcgccaagttccgggcgatccggcggctctgggcccgggtcgagcaggcctgcggcctcgagccgaagccgct
gcgggtcctggccgagaccgcgtggcggatgatgagccgccgtgacccattcgtgaacatcctgcgcaccaccatggcctcggcctccgc
cggcctcggcggggcggattcggtgaccgcgctgccctacaccctggcgctcggtctgcccgacgcctttgcccggcgggtggtgcgcaa
cagccagatcgtgctgatcgaggaatcgaacctcgccaaggtctccgatccggcggccggcgcgggcggcttcgaggcgctgaccaccg
aactgaccgagaaggcctgggcggccttccaagagatcgagcgagagggcgggatcgccgcgagcctggagtccggcgccttcgcggg
tcggatcgcggcggtggcgcaggcccgggcacgggcggtggcaacccgcaaggagccgctgaccggcgccagcgagttccccttcctc
gccgagaagccggtgacggtgctcgacgtggcgccgaaccccgccgcttcctcagagggggccttgccctcgcaacgcctcgccgaacc
ctacgaggccctgcgcgacgcctccgacgccgtccgcgagcggacgggcaagcgcccggcggtcttcctggccaatctcggtcccgtgg
cggttcacaatgcccgctcgaccttcgctgccaacgccttcgctgcgggtggcatcgaggccatcggcaatgacggcttctccgacatgcag
gccctggccgatgccttcaaggcgtcgggcgcgcggatcgcctgcctgtgctcgtccgacaccgtctaccagaccgaggccgtgcccgcg
gccgaggcactaaaggcggccggcgccggcacgatctacctcgccggcaaacccgccgaggccgaggctctgcgccaagcgggcgtc

gaaggcttcctgttcgccggttgcgacctgttggcgctcctgcgggaagcggccgagcgcgcgaagggctgactaacactcaaggccgcg
tgagcaaccttgacactgcgggacgcaagacgatcttccggctgaaacgggccgacacggccccatgccctgagccggatgtcatccccat
gcgccttccgatccttgccgtcgccacctttgccatcgccgtcggcagcgccggaacatcggaggcggccaagcgcaaggtgcaggtgcc
gcatcgctacgacggccgctggagcatcgaggtcgtgaccctcgacggcccctgcgaccgggcctatcgctacggcatccagattgaccgt
ggcggggcacgattgggattttgggcactcgacgcccgcgcgacgccccggcatgaaggggccatgagcgcgacccttcccgacatgga
cacactccgcgagcggcttctggccggcgaccgagcggcgctcgcccgcgccatcacgctggccgagtcgcgccgggccgatcaccgg
gcggcagtgcgcgacctcatcgacgcggtgctgccgcagacggggcgggcgatccgcgttggcatcaccggcgtgccgggcgtcggca
aatccaccaccatcgacgcgctcggctcgctcctgaccgcggcgggtcacaaggtggcggtgctcgccgtcgatccgtcctcgacccgcac
cggcggctcgatcctcggcgacaagacgcggatggcgcggctcgccatcgaccgcaacgccttcatccggccctcgccctcctcgggcac
gctcggcggcgtcgcggccaagacccgcgagaccatgctgctgtgcgaggcggcgggtttcgacgtgatcctggtggagaccgtcggcgt
cggccagtcggagacggcggtggccgatctcaccgactttttcctcgtgctgatgctgccgggcgccggcgatgagcttcagggcatcaag
aagggcattctcgaactcgccgacatgattgcggtcaacaaggccgacgatggcgacggcgagcggcgggcgagtgccgccgcctccga
gtaccgcgctgcgctccacatcctgacgccgccttccgccacctggacgccgccggtggtcacgatctcgggcctgcacggcaaggggct
cgattccctctggagccggatcgaggatcaccgcagcaagctcaccgcgaccggcgagatcgcgggcaagcgccgcgagcaggacgtg
aagtggatgtgggcgctcgttcacgagcggctgcaccagcgcctcgtcggcagcgccgaggtgcggcaggccaccgccgaggctgagc
gggcggttgcgggcggcgagcactcgcccgctgccggcgccgatgcgatcgcgaccctgatcgggttgtgactccaggcatcaaataaaa
cgaaaggctcagtcgaaagactgggcctttcgttttatctgttgtttgtcggtgaacgctctctactagagtcacactggctcaccttcgggtggg
cctttctgcgtttatacctagggcgttcggctgcggctggcgctgggcctgtttctggcgctgggcgtttaagggcaccaataactgccttaaaa
aaattacgccccgccctgccactcatcgcagtactgttgtaattcattaagcattctgccgacatggaagccatcacagacggcatgatgaacct
gaatcgccagcggcatcagcaccttgtcgccttgcgtataatatttgcccatggtgaaaacgggggcgaagaagttgtccatattggccacgtt
taaatcaaaactggtgaaactcacccagggattggctgagacgaaaaacatattctcaataaaccctttagggaaataggccaggttttcaccg
taacacgccacatcttgcgaatatatgtgtagaaactgccggaaatcgtcgtggtattcactccagagcgatgaaaacgtttcagtttgctcatgg
aaaacggtgtaacaagggtgaacactatcccatatcaccagctcaccgtctttcattgccatacggaattccggatgagcattcatcaggcggg
caagaatgtgaataaaggccggataaaacttgtgcttatttttctttacggtctttaaaaaggccgtaatatccagctgaacggtctggttataggt
acatt
